# From stigma to support: Assessing the impact of school mental health literacy program in Addis Ababa, Ethiopia

**DOI:** 10.1371/journal.pgph.0007153

**Published:** 2026-09-01

**Authors:** Lidia Veliaminova, Surafel Worku Megersa, Finina Abebe Ware, Paula Gerbig, Sandra Dehning, Eshetu Girma, Andrea Jobst

**Affiliations:** 1 Teaching and Training Unit, Division of Infectious Diseases and Tropical Medicine, LMU University Hospital Munich (LMU), Munich, Germany; 2 Center for International Health CIHLMU, LMU University Hospital, LMU Munich, Munich, Germany; 3 Department of Psychiatry, Saint Paul’s Hospital Millennium Medical College, Addis Ababa, Ethiopia; 4 Clinton Health Access Initiative-Ethiopia, Addis Ababa, Ethiopia; 5 Department of Psychiatry and Psychotherapy, LMU University Hospital, LMU Munich, Munich, Germany; 6 Department of Child and Adolescent Psychiatry, Psychosomatic and Psychotherapy, LMU University Hospital, LMU Munich, Munich, Germany; 7 African Population and Health Research Center, Nairobi, Kenya; Universiti Kuala Lumpur Royal College of Medicine Perak, MALAYSIA

## Abstract

Mental health stigma remains a major barrier due to limited mental health promotion and care among adolescents in low- and middle-income countries (LMICs), due to a lack of mental health literacy, sociocultural beliefs, and misconceptions which contribute to delayed help-seeking and poor mental health outcomes. In Ethiopia, stigma surrounding mental illness can result in social exclusion, discrimination, and abandonment by family members. This study evaluated the effectiveness of a culturally adapted, school-based mental health literacy and anti-stigma intervention among secondary school students in Addis Ababa, Ethiopia. The intervention was culturally adapted in collaboration with local mental health experts and adolescent students to ensure relevance to the Ethiopian context. Using a train-the-trainer approach, seven school nurses and health extension workers were trained to deliver the intervention to 186 students across four secondary schools. Implemented over one week, the intervention utilized three components: (1) an interactive mental health literacy curriculum delivered through PowerPoint presentations and participatory activities, (2) a contact-based session involving a person with lived experience of mental illness, and (3) mental health promotion materials, including posters and bookmarks displaying anti-stigma messages. Outcomes were assessed using self-administered questionnaires completed before the intervention and three weeks after its completion. Following the intervention, students demonstrated improved mental health knowledge and more positive attitudes toward mental illness (t(185) = 8.67, p < 0.001). Social distance, avoidance, and discomfort toward individuals with mental health problems were reduced, while intentions to seek help increased, with participants reporting greater willingness to seek support from formal and informal sources. These findings suggest that a culturally adapted, school-based mental health literacy and anti-stigma intervention was associated with improving adolescents’ mental health knowledge, reduce stigmatizing attitudes, and strengthening help-seeking intentions. Such interventions represent a promising strategy for supporting adolescent mental health and addressing stigma in Ethiopia and other LMIC settings.

## Introduction

Mental health disorders have a peak onset around age 15 years old, and by age 25, 63–75% of mental disorders will have already emerged [1]. Globally, one in seven adolescents aged 10–19 experiences a mental disorder [1], and 80% of people with a mental disorder live in low- and middle-income countries (LMICs) [2]. In Africa, one in seven children has experienced significant psychological challenge, and one in ten meets the criteria for a psychiatric diagnosis [3]. However, access to mental healthcare professionals in LMICs is limited, especially for those living in rural areas [4]. In Ethiopia, there are only 0.04 psychiatrists per 100,000 people [4].

From 2010 to 2021 the disability adjusted life years (DALYs) jumped to 16.4% for major depressive disorders and 16.7% for anxiety disorders [5]. Mental disorders have considerable economic consequences with 418 million DALYs contributing to an economic loss estimated at 5 trillion USD [6]. Therefore, early detection and treatment of mental health disorders at a young age are essential.

Stigmatization of individuals with mental health problems or disorders contributes to hesitation in seeking help, delaying early recognition and treatment, and severely impacting their well-being, self-esteem and social engagement [7]. Despite the lack of financial backing or insufficient policy cascading through LMICs healthcare systems, stigma is the fourth highest barrier when it comes to help-seeking in individuals suffering from mental-health disorders [7]. Stigma is defined as a social process that arises when labeling, stereotyping, separation, status loss, and discrimination occur together in a power situation which allows these processes to unfold [8]. There are varying levels of stigma against people with a mental health condition: self-stigma, public stigma, stigma by association, e.g., having a family member with a mental health condition, and structural stigma and discrimination [9]. Stigmatization of individuals with mental health disorders are highly prevalent in LMICs, often reinforced by cultural beliefs in contagion or supernatural causes [10] and it frequently prevents affected individuals from seeking help [11]. In Ethiopian culture, mental health disorders are often attributed to supernatural causes, such as evil spirits or curses [12] and people frequently prefer consulting traditional or religious healers. Participants, particularly from religious schools, reported that religious and supernatural explanations are more widely accepted and that mental disorders are believed to be the results of human actions, sins, curses and evil spirits and that solutions only emanate from religious practice [13]. Accordingly, Abera et al. found that most Ethiopian parents – particularly those with lower levels of education – believe in supernatural causes of children’s mental health disorders, and 92.7% would prefer to consult a traditional healer [14]. This preference has also been identified as the primary barrier children and their parents face in accessing mental health care in other African countries [15]. Stigma was the second major barrier, with 45% of parents reporting shame regarding their child’s mental health condition, followed by a lack of knowledge about mental health issues as the third major obstacle [15].

Early stigma interventions targeting young people at the typical age of onset of mental disorders can have the greatest positive impact on help-seeking behavior, as people are less likely to anticipate social rejection [16]. Thus, anti-stigma interventions may possess preventive potential. Moreover, anti-stigma interventions aim to promote greater social acceptance and integration of affected individuals, which in turn may enhance well-being and social connectedness, ultimately supporting rehabilitation from mental health disorders. Previous studies have shown that social contact-interventions – which facilitate interactions between individuals with a stigmatized condition and those without – are effective in reducing community-held stigmatizing beliefs about mental health [17]. Efforts have been made to implement anti-stigma interventions in Africa, but these have primarily focused on individuals with HIV or other stigma related health conditions [10]. Governments in LMICs often prioritize addressing immediate public health challenges, which diverts attention and resources away from stigma-related research. The lack of political support for addressing stigma and implementing anti-stigma interventions further hinders progress in these countries [18]. Some notable and impactful mental health stigma campaigns have been successfully conducted in HICs, such as the United Kingdom, Canada, and New Zealand [19]. Most of these anti-stigma intervention studies reported positive outcomes exceeding 82%, suggesting promise for similar initiatives in LMICs [10].

Schools have been found to be an effective setting for delivering mental health programs to students, and school-staff – particularly teachers – well-suited to implement them [20]. In a review-article by Yamaguchi et al (2020), 8 out of 10 studies focusing on teachers’ mental-health literacy reported significant improvement in stigmatizing attitudes. Most teachers are also gaining greater confidence in their ability to support students with mental health problems by mental-health literacy campaigns [21]. Other anti-stigma interventions have successfully been delivered by psychologists, healthcare staff, or trained graduate and undergraduate psychology students [22]. There is a potential for scaling up interventions to treat mental health problems in schools, which could help to increase young people’s access to care in settings where healthcare professionals are scarce by employing teachers or community health workers [23].

There is a limited amount of research and evidence on the successful implementation of mental health anti-stigma interventions in LMICs. Some studies have reported short-term benefit, such as positive changes of attitudes towards people with a mental health disorder [24]. Thornicroft highlights that social contact-based anti-stigma interventions have been the most successful in LMICs, as they appear to be the most effective in improving stigma-related knowledge and attitudes. There are research gaps in LMIC’s targeting culturally relevant mental health school-based interventions for adolescents [25]. Building on these findings, further research is needed to examine culturally adapted, contact-based anti-stigma interventions among adolescents in LMIC settings.

The purpose of our study is to examine whether a culturally-adapted week-long, school-based anti-stigma intervention for adolescents in Addis Ababa, Ethiopia, can reduce mental health stigma. We hypothesized that, following the intervention, mental health literacy, positive attitudes towards mental health, stigma awareness and actions taken to combat stigma might increase, while social distance, avoidance and discomfort when interacting with individuals with mental health conditions would decrease. Additionally, we expected a greater likelihood of help- seeking actions in the event of mental health disorders.

## Methods

### Ethics statement

Primary ethical approval was obtained from the Addis Ababa University Research Ethical Committee School of Public Health (Approval number 22-0919) and the LMU Munich Ethical Review Board consented to the secondary data analysis (Approval number 09-2025). Permission to conduct the intervention during school hours was also obtained from the four schools. After the researcher explained the purpose of the study to the study participants, assent was obtained from the schoolchildren and formal written informed consent was obtained from the parents, school nurses (SNs), and health extension workers (HEWs) prior to data collection.

### Study design

**Fig 1:**
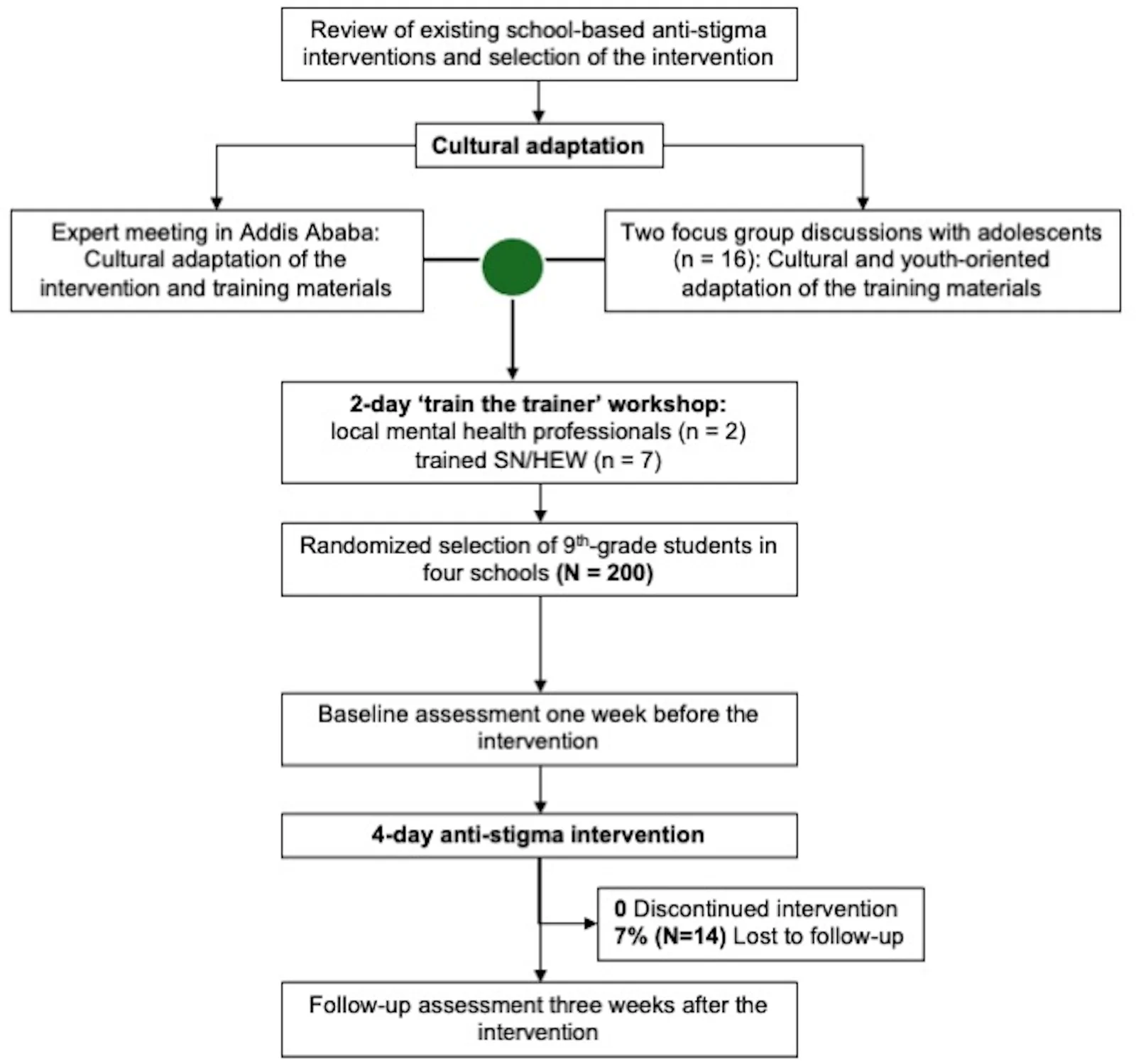
Study flow diagram of the cultural adaptation, participant recruitment, intervention, and follow-up process.

The anti-stigma intervention study design employed a pre-posttest quasi-experimental study without a parallel control group. The intervention manual was adapted from an original study by Link et al. (2020), conducted by a group of psychiatrists in Texas, United States. In the original study, a mental health anti-stigma curriculum was delivered to students by their teachers over the course of one week. This was supplemented with a contact-based component involving two young adults living with a mental disorder, as well as printed educational materials such as posters and bookmarks [26]. In the current study, the intervention was implemented in Addis Ababa following the same structure with adaptations to align with the Ethiopian cultural context (see Fig 1). SNs from private schools and HEWs from government schools were trained and supervised to deliver the anti-stigma intervention across selected high schools. The 2-day training was provided by two local mental health professionals from Addis Ababa (psychiatrist specialized in children and adolescents, and an M.Sc. in mental health). In addition, ongoing supervision during the intervention was provided to the SNs and HEWs by the psychiatrist and one public health professional to ensure consistency with the implementation of the program and data collection.

### Participants

The school nurses and HEW were recruited between 6^th^ of March, 2023 and trained for one week until 11^th^ of March, 2023. The school children were recruited and baseline evaluation was received on 20^th^ of March, 2023 and they received the intervention for one week. The second evaluation was done after 3 weeks on 17th April 2023.

To enroll the study participants, a list of schools was obtained from the Addis Ababa Health Bureau. From this list, four schools (two private and two public) were selected based on two criteria: first the school’s willingness to conduct the study and second, location due to proximity with the School of Public Health, which facilitated and supervised the study with the schools. Our student inclusion criteria: Being a regularly enrolled 9th grade student at one of the four selected schools during the 2022/2023 academic year, providing a written informed assent and, obtaining signed parental informed consent. The exclusion criteria: severe acute physical or cognitive illness on the days of data collection that precluded completion of the self-administered digital questionnaire. The samples were distributed proportionally between public and private schools according to the number of students in each school. Since we did not conduct a formal power calculation, and the sample size was determined primarily by practical feasibility, resource and logistical availability, this study should be considered exploratory in its nature.. We targeted a cluster layout of 50 students per school across the 4 school with distinct institutional environments (targeting 200 students in total) to ensure a sufficient sample for paired non-parametric and parametric analyses while maintaining operational oversight and operational fidelity. Subsequently, sections were chosen in a lottery method from each selected school. Finally, a list of students from the selected sections was prepared and participants were recruited.

The study population consisted of ninth-grade students from four selected high schools in Addis Ababa, who consented to participate in the anti-stigma intervention. Despite collecting a baseline data from 200 students across the four schools, at the 3-week post-intervention evaluation, 186 students successfully completed the follow-up assessment, representing a loss to follow-up 7% (n = 14). The loss to the follow-up resulted from students’ absenteeism on the day of the post-assessment or incomplete or missing data on the ODK application (see Fig 1). No formal statistical comparison analysis was performed between completes and non-completes. Because the proportion of missing post-intervention data was relatively small, and the primary objective of the study was to evaluate pre-post changes among participants who completed the intervention, therefore, a complete-case analysis was considered appropriate.

Two private schools (*Raguel and Radical*) and two governmental public schools (*Abune Basilios and Kokebe Tsibah*) were included. Each school had a planned sample of 50 students, and sections within the schools were randomly selected. The final list of participants was obtained after this selection process. All eligible students within the selected sections whose parents provided informed consent and who gave personal assent were invited to participate, targeting approximately 50 students per school to reach the planned sample. The overall mean age of participants was approximately 15 years. Specifically, students from Abune Basilios (n = 50) had a mean age of 15.7 years (SD = 1.1), from Kokebe Tsibah (n = 44) 15.7 years (SD = 1.5), from Raguel (n = 43) 14.8 years (SD = 0.7), and from Radical (n = 49) 14.7 years (SD = 0.8) (see Table 1). School type (private vs public) was analyzed as an independent variable potentially influencing intervention outcomes.

**Table 1:** Sample characteristics of students by school.

Sample characteristic	Abune Basilios (N = 50)		Kokebe Tsibah (N = 44)		Raguel (N = 43)		Radical (N = 49)	
Age (yrs): mean ± SD	15.7 ± 1.1		15.7 ± 1.5		14.8 ± 0.7		14.7 ± 0.8	
Gender								
Female (n/%)	30	60	32	72.7	29	67.4	14	28.6
Male (n,%)	20	40	12	27.3	14	32.6	35	71.4
Religion								
Orthodox (n, %)	31	62	41	93.2	41	95.3	41	83.7
Catholic (n, %)	10	20	1	2.3	2	4.7	1	2
Protestant (n, %)	9	18	2	4.5		NA	7	14.3
Living Arrangement								
With Parents (n, %)	31	62	24	54.5	36	83.7	37	75.5
With One Parent (n, %)	13	26	11	25	5	11.6	6	12.2
With Other Relatives (n, %)	6	12	9	20.5	2	4.7	6	12.2

The study also involved two young adults with a mental disorder, one male and one female who were recruited to participate in our contact-based session with the adolescents. They were recruited via purposive convenience sampling through clinical networks at a tertiary psychiatric unit in Addis Ababa. They were clinically stable, well-functioning, undergoing regular follow-up and deemed emotionally and psychologically well enough by their treating psychiatrists to participate in a public speaking session to the students. Their participation was completely voluntary. Formal written consent was obtained from both of them. They received detailed briefing on the school environment and main areas. They were compensated for their time and transportation.

In addition, two health staff (one female and one male) were recruited from each school to deliver the intervention – school nurses (SNs) from private schools and health extension workers (HEWs) from governmental schools. Since both SNs and HEWs provide comparable health services within their respective school system, they were considered functionally equivalent for this study. All selected staff had at minimum one year of working experience, resulting in a total of seven SNs/HEWs who were trained to implement the anti-stigma intervention at the schools. There was a total of (n = 7) SN and HEW who had an average age of 37.29 years (SD = 11.22), and an average of 8.14 years of working experience (SD = 7.56), 5 of the staff had a Bachelor of Science and 2 had a Master’s of Science.

### Data collection instruments and outcome measures

The students completed sociodemographic information questionnaire package – one week before and three weeks after the intervention. Data were collected using Open Data Kit (ODK), a mobile phone application that enabled digital recording and processing of pseudonymized data.

This included the *Knowledge and Positive Attitudes Scale* [27] in an adapted version by Link et al (2020). The 21-item scale assesses mental health literacy and attitudes towards mental health issues using a 5-point Likert scale (1 = strongly agree to 5 = strongly disagree). In our study, the scale demonstrated a Cronbach alpha (α) of 0.63. Example items include: *“Most people with severe mental illness do not get better, even with treatment,” or “it would be embarrassing to have a mental illness.”* Negatively worded items were reverse scored during the analysis.

The *Stigma Awareness and Action Scale* measures participants’ observations of stigma-related actions in the community or school setting, as well as their own taken actions against stigma. It is an 8-item binary scale (yes/no) with a Cronbach alpha (α) of 0.63 in our study. Example items include: *“I heard people use slang terms about mental illness like “psycho,” “crazy” or “looney” to put people down,”* or *“I told somebody it is wrong to make fun of a person with mental illness.”*

The *Avoidance and Discomfort Scale* assesses avoidance behaviors towards individuals with mental health disorders and the level of discomfort experienced in their presence. It consists of 6 binary items (yes/no), such as*: “I avoided a kid who was depressed and never wanted to do anything fun”,* or *“I felt uncomfortable around a kid in my class who has a mental health problem.”* In this study, the scale demonstrated a Cronbach alpha (α) of 0.67.

The *Social Distance Scale* is a 6-item Likert measure (1 = definitely yes to 4 = definitely no; α = 0.86) that assesses the extent to which youth are unwilling to interact with someone with a mental illness (e.g., “*Would it be okay with you to invite somebody with mental illness to your home?”*) Higher scores indicate a stronger desire for social distance.

The *Personal Help-seeking Behavior scale* consists of 7 items assessing the likelihood of seeking help from different sources (friends, parents, a doctor, a priest or religious healer, a school counselor, a therapist or counselor outside school, and by considering taking medication) in the event of a mental health problem. Example items include: *“I would talk to my parents if I were having a mental health problem”*. In this study, the scale demonstrated a Cronbach alpha (α) of 0.76.

All scales were translated into Amharic and then back translated to check its consistency. This was followed by a qualitative content validation via our youth focus groups during the adaptation phase. However, a formal psychometric validation study of these scales within the Ethiopian context has not been published yet.

### Intervention structure and components

The intervention was held during school hours. The SNs/HEWs from each school were delivering the anti-stigma intervention to the students. Facilitators underwent a mandatory 2-day intensive training workshop covering adolescent mental health, anti-stigma strategies, crisis mitigation protocol and digital data collection tracking via ODK. The intervention, adapted from the original program *(Eliminating the Stigma of Differences – ESD)* by Link et al. (2020), consisted of three components: (1) classroom training sessions delivered by school nurses (SNs) or health extension workers (HEWs) to the students using PowerPoint presentations and interactive activities (e.g., writing a letter to a person with lived experience), (2) a contact-based session with two young adults aged 20–25, one male and one female living with a mental disorder, and (3) distribution of printed information materials (posters and bookmarks). The original program by Link at al. (2020) was delivered to schoolchildren with a mean age of 11.5 years. In this study, we chose to include slightly older children – adolescents – due to the peak incidence of mental health disorder onset during this developmental period. Adolescence is a critical stage of development; therefore, an intervention during this period may promote health-enhancing behaviors that persist into adulthood [28].

The training sessions took place over four days, with one 30 – 50-minute session per day integrated into the students’ school timetable. Students were also offered refreshments during the sessions. The first three sessions consisted of classroom training, while on the final day, students participated in a contact-based session with individuals with lived experience of mental health disorders. Classroom training sessions delivered by SNs/HEWs included PowerPoint lectures on varying mental health topics such as stigma and discrimination, common mental disorders: anxiety, depression, psychosis, and attention deficit hyperactivity disorder, with their etiology and their manifestations. Students were also shown options for treatment, and recommendations for better mental health. They were also given information about the available mental health providers in the country. Group discussions, contact strategies with persons with lived experience, small role-plays, and writing a letter to an individual with a lived experience of mental health problems were also part of the week-long intervention curriculum. During the classroom training, students were asked to write an anonymous, brief letter expressing their thoughts, support, or questions to a hypothetical peer or individual experiencing a mental health struggle. The primary educational objective was to foster empathy and perspective-taking. Examples of what students typically wrote included messages of solidarity (e.g., “I want you to know that you are not alone and having a mental illness does not change your value”) or a newly gained awareness (e.g., “I used to think that people could just choose to stop being depressed, but now I understand it’s a medical condition and I want to support you”). To ensure participants confidentiality, the following steps were strictly enforced: 1. Anonymity: students were explicitly instructed not to write their names, section numbers, or any identifying markers on the letters. 2.Voluntary participation: Students were informed that writing the letter was a reflective learning exercise, and they could choose not to submit their letters to the facilitators if they felt uncomfortable. 3. Data handling: The letters were collected, and reviewed by only the facilitators and they were not shared publicly or linked to the quantitative ODK survey data. Finally, they were securely discarded after the project completion.

Printed educational materials – posters and bookmarks – were provided throughout the week-long anti-stigma intervention. Posters were displayed in highly visible locations on the school campus, such as the library, and bookmarks were given to students to use and take home. These materials featured anti-stigma slogans, such as *“I am not my mental illness.”*

The contact-based session involved one male and one female young adult with lived experience of a mental disorder. This age group was purposefully selected so that the contact-based session featured individuals close in generation to our adolescent participants, making their lived experience more relatable. They shared their personal experiences, including the onset and progression of symptoms, hospitalizations and treatments, and coping strategies. At the end of the session, the SN or HEW facilitated an interactive question-and-answer segment between the students and the individuals with lived experience. When mental health concepts arose during the discussion, the trainers provided additional explanations as needed to ensure clarity, including information on the broader impact of mental health problems on social relationships, academic performance, and professional life.

The posters and bookmarks featured images of athletes and mental health slogans, such as “*People with mental illness can be productive members of society”*, along with other destigmatizing messages regarding mental illness. They also included resources, such as phone numbers and information on where to seek help for mental health problems.

### Cultural adaptation steps for intervention package development

A unique aspect of this study was its cultural adaptation. All materials utilized in this study were adapted to ensure cultural relevance and accessibility for all participants. We based our adaptation process on the *10-step Mental Health Cultural Adaptation and Contextualization for Implementation (mhCACI)* framework proposed by Sangraula et al. (2021) and conducted five key adaptation steps: First, a workshop was held in Addis Ababa with mental health experts from Ethiopia and Germany, who jointly reviewed, discussed and adapted the original training materials from Link et al. (2020), focusing on language, content, and replacing pictures with culturally appropriate alternatives. Second, a local qualitative assessment was conducted on the topic, which will be published separately. Third, all training materials were translated into Amharic, the local language, and reviewed by independent local professionals. Fourth, focus group discussions were led by a public health professional with school-aged youth of similar age (around 15 years old) – one group of eight randomly selected female students and another of eight randomly selected male students. This step reflects the participatory approach of our study. During these discussions participants were provided with the pre-adapted printed training materials (bookmarks and posters) and were invited to modify them to better align with their preferences, cultural context and youth perspective (final version of the printed materials see supplement 1).

### Statistical analysis

Sociodemographic and pre- and post-test questionnaire data were compiled and merged into an Excel file. The data were then cleaned and analyzed using R (Version 4.4.2) employing a range of statistical methods including paired t-tests, Wilcoxon signed-rank test, and linear regression. Shapiro-Wilk tests were performed to assess the normal distribution of the data. The significance level was set at α = 0.05, predetermined prior to analysis and p-value reporting.

For the Likert-type scales – the *Knowledge and Attitudes Scale,* a paired t-test was used to assess changes in responses between pre- and post-test of *Knowledge and Attitudes Scale*. Mean scores, mean difference and standard deviations were calculated. To account for multiple testing, Benjamini-Hochberg false discovery rate (FDR)-adjusted p-values were reported. The *Social Distance Scale* was analyzed using a Wilcoxon-signed rank test due to non-normality despite its Likert-type scale, as assessed by the Shapiro-Wilk test.

The binary-response questionnaires the *Stigma Awareness and Action Scale*, the *Avoidance and Discomfort Scale*, and the *Personal Help-Seeking Scale* were analyzed using the Wilcoxon signed-rank test, a non-parametric method appropriate for paired data. For each scale, individual binary items were summed to create participant-level total scores; because these summed scores are ordinal, bounded, and not normally distributed, pre- and post-intervention differences were analyzed using the Wilcoxon signed-rank test rather than a parametric alternative. We calculated the mean number of pre- and post-intervention “yes” responses and their changes for each scale. Both raw and adjusted p-values were calculated, with the Benjamini-Hochberg procedure applied to the raw p-values to control the false discovery rate (FDR).

## Results

### Key pre-post intervention measurement results

**Fig 2:**
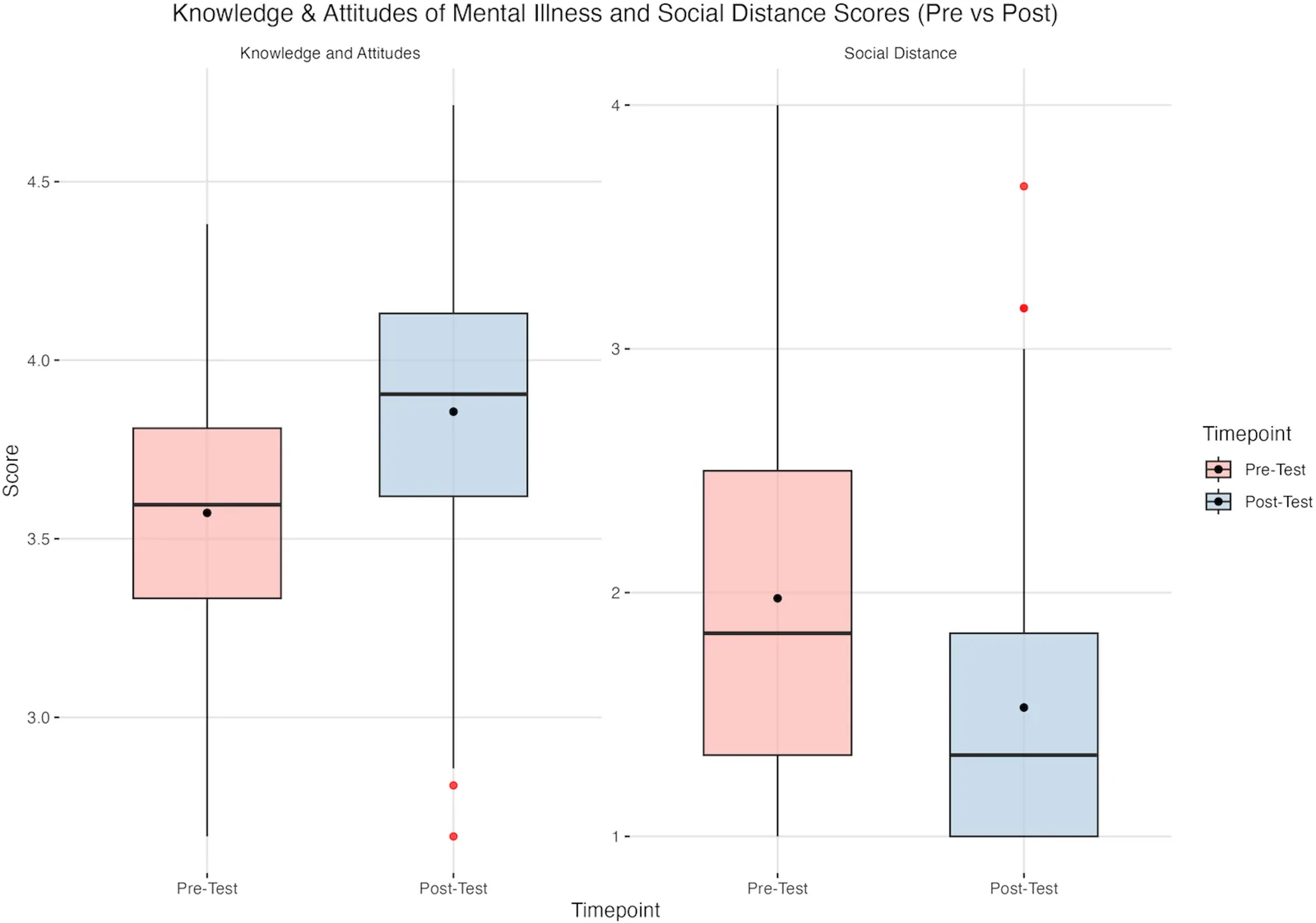
Boxplot of knowledge and attitudes of mental illness and social distance scores. Note: Boxplot of the pre and posttest results from Knowledge and Attitudes Scale compared to Social Distance Scale. Red points are represented by data outliers.

The pre- and post-test scores on the *Knowledge and Attitudes Scale* (Table 2) showed a mean increase of 0.28. The paired t-test was significant, *t*(185)=8.67, *p* < 0.001. The 95% confidence interval [0.22,0.35] further supports the robustness of this statistically significant result (see Fig 2).

**Table 2:** Test statistics of paired t-tests. N = 186 (N = total number of student participants from all four schools).

	Mean pre	Mean post	Mean difference	$SD_{diff}$	*T*	*df*	*p value (raw)*	*Adjusted* *p value*	95% CI	Cohen’s *d*
Knowledge and Attitude’s Scale	3.57	3.86	0.28	0.45	8.67	185	<0.001	<0.001	[0.22,0.35]	0.64

*Note* Mean difference indicates Post – Pretest value. SD_diff = Standard Deviation of the Differences. T = t-statistic. df = degrees of freedom. CI = 95% Confidence Interval [lower,upper], Cohen’s d = effect size. All numerical values are rounded to two decimal places. *p < 0.05; **p < 0.01; ***p < 0.001 significance level. *Adjusted p-value was corrected for multiple testing using the Benjamini-Hochberg (FDR) procedure.

### Association between knowledge and attitudes and social distance

We specifically aimed to predict post-intervention social distance, which showed the greatest improvement, using baseline Social Distance and Knowledge and Attitudes. To this end, we applied a linear regression model (Table 3). The model included pre-intervention Knowledge and Attitudes scores, baseline Social Distance scores and school affiliations as the independent variables, with post-intervention social distance as the dependent variable. School affiliation is operationalized as a categorical dummy-coded covariate indicating which of the four participating high schools (school 1, school 2, school 3, or school 4) a student was enrolled in. This accounts for structural, environmental, and socio-demographic variations across private and public-school settings*.* Multicollinearity among predictors was assessed using variance inflation factors (VIFs) and tolerance values. All VIFs were low (adjusted GVIF range: 1.02-1.23) and all tolerance values exceeded conventional thresholds (0.66-0.96), indicating no problematic multicollinearity. Assessment of residual normality using both a histogram (showing a bell shaped-curve) and a Q-Q plot (showing an approximately straight line) indicated that the assumption of normality was met, supporting the use of the linear regression model.

**Table 3:** Baseline-adjusted model predicting social distance outcomes.

Predictor	Coefficient 𝐁	SE	*t*	p value
**Participants (N = 186)**				
Intercept	0.818	0.472	1.735	0.084
Social Distance (Pre)	0.345	0.057	6.104	<0.001
Knowledge and Attitudes (Pre)	-0.046	0.114	-0.408	0.684
School 2 pre:	0.416	0.103	4.034	<0.001
School 3 pre:	0.360	0.098	3.654	<0.001
School 4 pre:	-0.002	0.103	-0.019	0.985

*Note:* Model Summary: R^2^ = 0.267, Adjusted R^2^ = 0.247, Residual SE = 0.485, *F* = F-statistic with respective degrees of freedom (*df*1, *df*2), *F*(5, 180) = 13.14, *p* < 0.001, Statistical significance: *p < .05, **p < .01, *p* *** < .001. *Note: Social Distance used as dependent variable. School 2,3,4 were tested with school 1 as reference with baseline (pre-test) social distance used as covariate.

Baseline Social Distance scores were a significant predictor of post-intervention Social Distance scores (*B* = 0.345, SE = 0.057, *t* = 6.104, *p* < 0.001) (Table 3). The positive coefficient indicates that for every one-unit increase in baseline Social Distance, post-intervention Social Distance increased by 0.345 units, holding other predictors constant. After accounting for baseline Social Distance, pre-intervention Knowledge and Attitudes scores were not a significant predictor of post-intervention Social Distance scores (*B* = -0.046, SE = 0.114, *t* = -0.408, *p* = 0.684). In *t*he linear regression model, School 1 was used as the reference category for the other three schools. The results indicated that School 2 (*B* = 0.416, SE = 0.103, *t* = 4.034, *p* < 0.001) and School 3 (*B* = 0.360, SE = 0.098, *t* = 3.654, *p* < 0.001) were significant predic*t*ors, showing higher post-intervention Social Distance scores relative to School 1, while School 4 (*B* = -0.002, SE = 0.103, t = -0.019, *p* = 0.985) was not a significant predictor.

In summary, the linear regression model identified baseline Social Distance as the strongest significant predictor of post-intervention Social Distance scores, while pre-intervention Knowledge and Attitudes was not significantly associated with post-intervention Social Distance once baseline levels were accounted for. The intercept was not statistically significant (*B* = 0.818, *t* = 1.735, *p* = 0.084), indicating that when all predictors were set to zero, the expected post-intervention Social Distance score did not differ significantly from zero. Overall, school affiliation emerged as a significant predictor for two of the three comparison schools, suggesting that school affiliation was associated with meaningful differences in post-intervention Social Distance scores, with Schools 2 and 3 showing higher scores relative to School 1.

The *Stigma Awareness and Action Scale* showed a median increase of one unit, rising from 2 pre-intervention to 3 post-intervention, out of a maximum of 8 possible `yes` responses (Table 4). The Wilcoxon-signed rank test yielded *W* = 5956.5, *Z* = 2.47, *p* = 0.012 (after correction for multiple testing), indicating a statistically significant improvement. The overall effect size was small (|*r*| = 0.21).

**Table 4:** Test statistics from the Wilcoxon signed-rank tests across outcome measures.

	Median pre	Median post	Wilcoxon W	Z	Raw p (two-tailed)	Adjusted p (FDR)	|r| Effect size
Stigma Awareness and Action Scale	2	3	5956.5	2.47	0.012	0.012	0.21
Avoidance and Discomfort Scale	1	0	2003.0	-4.26	<0.001	<0.001	-0.39
Personal Help-Seeking Scale	6	7	4921.0	3.18	0.001	0.002	0.29
Social Distance Scale	4	2	6897.0	5.91	<0.001	<0.001	0.52

*Note:* Wilcoxon Signed-Rank Tests were used to compare pre-and post-intervention scores. P-values were adjusted using the Benjamini-Hochberg False Discovery Rate (FDR) procedure. *p < .05, **p < .01, ***p < .001. Median Difference*= (Median Posttest Score) - (Median Pretest Score). *W* = Wilcoxon test statistic. *Z* = standardized test statistic from Wilcoxon test. r = Z/√N, where N is the number of paired observations; *|r|* = effect size with ∣0.2∣ as small, ∣0.5∣ as medium, and ∣0.8∣ as large effect, per Cohen’s convention.

For the *Avoidance and Discomfort Scale*, the median score decreased from 1 pre-intervention to 0 post-intervention, out of a total of 6 possible `yes` responses. After correction for multiple testing, both the raw and adjusted p-values were *p* < 0.001, indicating a highly significant post-intervention effect. The Wilcoxon-signed rank test yielded *W* = 2003.0 and a standardized test statistic of *Z* = -4.26. The corresponding effect size (|*r*| = -0.39) indicated a medium effect, reflecting a notable reduction in avoidance and discomfort following the intervention.

In the *Personal Help-Seeking Scale*, which assessed personal help-seeking behaviors, the median score increased from 6 pre-intervention to 7 post-intervention, out of 7 possible ‘yes’ responses (*W* = 4921.0, *Z* = 3.18, *p* = 0.001), indicating a statistically significant improvement. The effect size (|r| = 0.29) suggest a small but meaningful impact of the intervention on this scale.

The *Social Distance Scale* demonstrated the most pronounced change among the measures, with the median score decreasing from 4 (out of 6 possible `yes` responses) pre-intervention to a 2 post-intervention (W = 6897.0, Z = 5.91, p=<0.001). This represents a highly statistically significant effect, with a medium effect-size (|r| = 0.52).

## Discussion

The study aimed to evaluate the potential of a school-based anti-stigma intervention in reducing mental health-related stigma, lowering barriers and increasing help-seeking behavior among adolescents in Addis Ababa, Ethiopia. To date, most stigma related interventions have been conducted primarily in high-income countries (HICs), where they have demonstrated significant changes in attitudes and practices following educational programs or interventions [29]. We used a culturally adapted version of a school-based anti-stigma program – *Eliminating the Stigma of Differences (ESD)* – originally developed by Link et al. (2020). This intervention was applied in 16 schools in Texas, United States with 416 schoolchildren in a randomized controlled study to investigate the effect of different components and the overall program. The authors found that the intervention was more effective than both a control intervention and a no-intervention group at increasing of knowledge, promoting positive attitudes, and reducing social distance. In the present study, we provide preliminary, promising results of a similarly adapted program in a low- and middle-income country (LMIC).

Our primary findings suggest that the anti-stigma intervention was feasible and associated with improved mental health literacy and more positive attitudes towards mental health, as reflected by the *Knowledge and Attitudes Scale*. Furthermore, participants demonstrated increased stigma awareness and promoting a greater willingness to take action to combat stigma within their communities or school environments, as measured by the *Stigma Awareness and Action Scale*. Our findings are consistent with the assumption that school-based anti-stigma programs and mental health awareness campaigns may have a positive impact on mental health literacy and awareness. In our study, students became more aware of their own and others’ stigmatizing behaviors, such as excluding a peer with a mental health problem from activities. Data collection was conducted by SN and HEW rather than by classroom teachers or school administrators, and all data were collected anonymously, thereby reducing the likelihood that students would feel pressure to provide responses perceived as socially desirable or institutionally expected.

Following the intervention, students were more likely to recognize such behaviors and showed greater willingness to take actions to combat stigma. These results point to an increased awareness of stigmatizing attitudes and behaviors in both oneself and others, which may contribute to reductions in stigma and more mindful interactions with individuals experiencing a mental illness. Overall, these changes suggest that students were beginning to develop a greater ability to recognize and critically reflect on stigmatizing attitudes and behaviors.

Second, we observed a significant post-intervention reduction in desired social distance toward individuals with mental health problems, as measured by the *Social Distance Scale*, as well as a decrease in avoidance and discomfort, as assessed by the *Avoidance and Discomfort Scale*. After the intervention, students appeared more willing to engage with a person with mental illness, reflecting a shift toward more inclusive social behavior. Interestingly, the regression analysis identified baseline social distance as the strongest predictor of post-intervention social distance, whereas baseline knowledge and attitudes were not independently associated with post-intervention social distance after adjustment for baseline social distance. This finding is consistent with theoretical models of stigma, which conceptualize knowledge, attitudes, and social distance as related but distinct constructs [1]. It suggests that initial levels of social distance may represent the most robust determinant of subsequent stigmatizing behavior, whereas the influence of knowledge and attitudes may operate indirectly or be largely captured by baseline social distance. In addition, school affiliation emerged as an independent predictor, indicating that contextual school-level factors may also influence intervention outcomes. Together, these findings suggest that reducing social distance may require not only improvements in mental health literacy but also interventions that directly address behavioral aspects of stigma while considering the broader school environment. Additionally, avoidance behaviors and experienced discomfort when interacting with peers and individuals with mental health problems showed a decrease post-intervention as measured by the *Avoidance and Discomfort Scale*. However, students already exhibited low pre-intervention Avoidance and Discomfort scores (mean = 1 out of 6), which further decreased to zero after the intervention. These findings suggest that, following the intervention, students may have become more comfortable interacting with individuals experiencing mental disorder. The pre-intervention low levels of Avoidance and Discomfort might have different reasons. One suggestion might be that while mental health stigma may exist in the broader Ethiopian society, younger populations, particularly school-aged children, who may be less influenced by societal stereotypes and more accepting of individual differences. Moreover, schools often promote cooperation, group activities, and peer interaction. Therefore, school students may naturally develop better comfort interacting with diverse peers.

In addition, associated improvements were observed in the students’ intention to seek help in the event of mental health problems as measured by the *Personal Help-Seeking Scale*. In Link et al.’s original study, students who took part in the curriculum reported substantially greater willingness to seek treatment for their mental health problems from different resources compared to the control group [26]. The students already exhibited high help-seeking behavior prior to the intervention, and post-intervention measurements indicate that the students might be more comfortable seeking help for their mental health, potentially suggesting a ceiling effect. 91.9% of students preferred to speak to a doctor, 90.3% to a parent, and 88.7% to a therapist. School counselor were the last preferred source of support, with 73.7% of students indicating that they would seek help form one. When directly compared, students in our study were more likely to seek help from a medical professional than from a priest, with only 82.3% indicating that they would turn to a priest. Despite studies showing parents prefer to go to a traditional healer in regards to mental illness, these students would prefer to see a doctor. At a traditional holy water site in Ethiopia, most help-seeking respondents preferred religious leaders or holy water sprinkling and believe the special healing properties of these traditional treatments [30]. One important consideration is that the students lived in Addis Ababa, the capital of Ethiopia, where access to modern healthcare is substantially greater than in rural areas of the country. Therefore, it should be noted that our study included only ninth-grade students from four selected school in Addis Ababa that agreed to participate. Consequently, the generalizability of our findings to rural regions of Ethiopia, younger or older adolescent, or out-of-school youth is limited and warrants further investigation in future research trials.

When comparing results from our study to the original by Link et al. (2020) from a Western and high-income setting, our findings in a low-resource setting similarly replicate the primary outcome findings reported by Link et al. In their study, school children showed greater knowledge and more positive attitudes, as well as reduced social distance, compared to children in the control groups [26]. These findings were attributed to the success of the curriculum during the intervention. However, they found no significant effects from the contact-based intervention or from printed materials alone. This is surprising, as a review by Thornicroft et al. found that social-contact based interventions with students achieved the greatest short-term attitudinal change in reducing mental illness stigma compared to other intervention components [24]. Our study, which incorporated all three components: curriculum, contact and materials, was associated with a medium effect (Cohen’s D = 0.64) on the Knowledge and Attitudes Scale, compared to Link et al.’s increase in the Knowledge and Attitudes Scale (Cohen’s D = 0.35). Thus, our findings similarly result in the primary outcome findings reported by Link at al. (2020) while in a different setting within the Global South and a LMIC context.

Our school-based educational anti-stigma intervention results also align with similar findings from other global studies. For example, a study using a similar school-based mental health intervention in Australia reported improvements in mental health literacy and reductions in stigma [31] and a school-based study conducted in Vietnam and Cambodia, which also employed a ‘train-the-trainer’ approach for teachers delivering a mental health stigma intervention, found lower stigma and higher mental health literacy among students who participated in the intervention compared to the control group [32]. Similarly, other studies examining knowledge, attitudes and willingness to interact with individuals with mental illness reported comparable patterns among students who received mental health instruction versus those who did not [27].

Mental health stigma remains a global health challenge rooted in societal and cultural factors and is one of the major barriers preventing people from seeking help [15]. In Ethiopia, where this study was conducted, limited mental health services further delays treatment, particularly among individuals without formal education or employment [33]. Mental health service implementation and funding remain very low in LMICs, highlighting the need for capacity building to reduce stigma and improve mental health literacy, ultimately encouraging people to seek help within the healthcare system. Mental health awareness interventions show particular promise in the adolescents age group and the school setting is an ideal environment to deliver training to entire age cohort of youth. A Training-of-Trainers (ToT) approach for school nurses or teachers may strengthen mental health education for both educators and students. However, mental health is rarely prioritized in LMICs’ healthcare budgets, and a severe shortage of professionals persist – for example, Ethiopia has only 0.08 psychiatrists per 100,000 people [34].

Considering this critical shortage of mental health professionals in low- and middle-income countries like Ethiopia, a training-of-trainers (ToT) approach combined with continuous capacity-building sessions to the key personnel among the school community – including teachers, school nurses, and school psychologists may play a vital role in integrating and scaling up school- based mental health interventions. Trained personnel should also receive an ongoing support and supervision from mental health experts such as psychologists and psychiatrists. To ensure adequate funding, effectiveness, and sustainability, collaborative efforts among the Ministry of Health, the Ministry of Education, international non-governmental organizations focusing on child and adolescent health, and other stakeholders are essential.

In addition, linking school-based interventions to the broader healthcare system may be crucial for establishing regular screening, referral pathways, and case management. As health extension workers (HEWs) are the integral part of the Ethiopian healthcare system, they may serve as the bridge between schools and clinics, assisting with follow-ups and providing community-level support. The expansion of school-based stigma intervention across Ethiopia would require stronger advocacy supported by evidence-based studies, engagement with local communities, and the facilitation of contact-based interventions. Securing funding and scaling up programs are essential priorities for integrating mental health initiatives into schools. Implementation may be strengthened through community outreach, training for teachers and school nurses, and collaboration with local psychiatrists and medical students. Building community partnerships will be critical for long-term success of school-based mental health programs aimed at reducing stigma.

### Strengths and limitations

A strength of our study is the structured cultural adaptation process, which followed five key steps outlined in the study design using the 10-step mhCACI. Cultural adaptation may play a crucial role in the development and successful implementation of study protocols, particularly in the context of mental health research [35]. In the African context, mental health studies addressing suicidal ideation, depression, or trauma have applied cultural adaptation using frameworks previously developed, e.g., in the US. These studies suggest promising implementation through lay health workers who support the communities in mental health matters and may help address the treatment gap in Africa [36].

The cultural adaptation process appears crucial for mental health interventions in the Global South, particularly anti-stigma, literacy and awareness programs, as most interventions are designed and validated in Western contexts. In LMICs, cultural adaptation is particularly important because concepts of mental illness, explanatory models, and help-seeking behaviors are shaped by local cultural and spiritual belief systems, which often differ substantially from the assumptions underlying Western intervention models. Without adaptation, interventions may run the risk of being perceived as irrelevant or even culturally inappropriate. Challenges encountered in implementing this type of intervention in a school-based setting stemmed from deeply rooted cultural stigma, low levels of mental health awareness and the widespread belief that mental illness is attributed to supernatural causes. These beliefs are embedded within the Ethiopian cultural and societal perspectives and became evident as the intervention was taking place. Transferring these interventions to other cultural contexts without clearly defined local needs and expectations can cause more harm than good. Therefore, cultural adaptation may bridge the gap between evidence-based mental health practices and local realities, potentially maximizing engagement, effectiveness, and long-term impact. Drawing on the experiences of local experts and youth focus groups may provide valuable insight for future local mental health professionals, helping them to better incorporate community perspectives into the design of future interventions.

Moreover, we applied patient and public involvement (PPI) by conducting focus groups with school youth to review and modify the training materials, as outlined in the study design. The local youth focus groups assisted us in co-designing the anti-stigma materials used throughout the study. Youth-focused participatory research may provide young people with developmental opportunities and foster a sense of contribution to their community [28]. Since the adoption of the United Nations Convention for the Rights of the Child, young people’s participation in research and decision-making has become a global priority. A survey conducted in five countries asked youth about their aspirations for engagement in mental health advocacy. Most of the 31,371 participants expressed a desire to be involved in mental health projects or support peers in low- and middle-income countries (LMICs) [37]. This participatory model ensures contextual safety and relevance and can serve as a replicable blueprint for scaling public health interventions across other LMIC settings. Such youth participatory research and interventions show promise in potentially improving students’ future mental health outcomes.

There are six major limitations to this study. First, there was no long-term follow-up beyond the initial post-intervention questionnaire, which was administered three weeks after the intervention. In contrast, the original study by Link et al. (2020) included a two-year follow-up period, which reported sustained effects. Due to limited resources, we were unable to obtain follow-up data. Thus, this study only captured short-term associations and cannot speak on long-term sustained outcome, resulting, in a lack of long-term changes in knowledge, attitudes, stigma, or personal help-seeking behaviors among students. Future studies should aim for a longer assessment period and long-term follow-up to evaluate the sustainability of intervention effects.

Second, our Cronbach Alpha value 0.63 for the Knowledge and Positive Attitudes scales and Stigma Awareness and Action scale indicated relatively modest consistency. This may reflect several factors, including the cross-cultural adaptation of psychological instruments originally developed and validated in Western settings for use among Ethiopian adolescents, the use of binary response formats for some instruments, which generally yield lower reliability estimates than Likert-type scales, and the multidimensional nature of stigma-related constructs encompassing related but distinct domains such as knowledge, attitudes, avoidance, and social distance. Despite these modest reliability coefficients, the scales were sufficiently sensitive to detect pre-post changes following the intervention. Nevertheless, future studies should include comprehensive psychometric validation and, where appropriate, further refinement of item wording to optimize the measurement properties of the Amharic versions for Ethiopian adolescents.

Third, this study did not employ a randomized controlled design and therefore lacked a control group. Consequently, it is not possible to determine whether the observed changes can be attributed solely to the intervention rather than to concurrent factors such as maturation testing effects, regression to the mean, or other temporal influences. Randomized controlled trials are needed to establish the efficacy of the intervention and strengthen causal inferences regarding its effects.

Fourth, the study design did not allow for disentangling the individual effects of the curriculum, printed educational materials, and the contact-based session. Therefore, it remains unclear which component contributed most to the observed improvements. Moreover, potential response bias, including desirability bias, may have led participants to overreported help-seeking intentions and endorse more socially desirable attitudes. Future studies should therefore incorporate objective behavioral measures.

Fifth, no formal power calculation was performed in the study, therefore it should be considered as explanatory and as a pilot in nature.

Lastly, students were nested within four participating schools, and responses from students within the same school may therefore not have been statistically independent. Although school affiliation was included as a fixed covariate to account for between-school differences, this approach does not fully address within-school clustering, which may have resulted in underestimated standard errors. Multilevel modelling was not feasible because only four schools participated, providing too few clusters for reliable estimation of random effects. Consequently, the statistical significance of the findings should be interpreted with appropriate caution.

## Conclusion

Mental health stigma is a global public health issue affecting people worldwide, particularly those in LMICs, where limited infrastructure, weak policy support, and low public awareness are further compounded by cultural beliefs on contagion or supernatural causes. This study provides preliminary evidence suggesting that a school-based anti-stigma program among adolescents was associated with reductions in stigma towards individuals with mental health disorders and associated improvements in mental health knowledge and help-seeking behavior among adolescents in Addis Ababa, Ethiopia. Future initiatives should focus on advocating for mental health awareness within the school system through educational, school-based anti-stigma intervention. Such interventions may enhance mental health knowledge, reduce stigma and potentially support long-term outcomes for adolescents in adulthood by promoting early detection and treatment through an increased likelihood of potentially seeking help. Mental health education may also contribute to economic benefits by promoting well-being, encouraging treatment-seeking, and fostering societal acceptance of individuals with mental health disorders. Although structural and deeply rooted societal barriers to mental health stigma remains; engaging youth in mental health initiatives shows promising potential for contributing to improved mental health globally.

## Supporting information

S1 Fig Posters and bookmarks created for the study. Visual materials designed and culturally adapted for the intervention by local psychologists featuring positive mental health quotes.
(TIFF)

S1 Data Study dataset. De-identified dataset containing the questionnaire responses collected for this study. All participant information has been anonymized to protect confidentiality.
(XLSX)

S1 Text Inclusivity in Global Research questionnaire. Completed PLOS questionnaire submitted in accordance with the journal’s inclusivity in global research reporting requirements.
(DOCX)
