# Supplementary material for: From stigma to support: Assessing the impact of school mental health literacy program in Addis Ababa, Ethiopia
Source: PLOS Glob Public Health. 2026 Sep 1;6(9):e0007153. doi: 10.1371/journal.pgph.0007153 (PMC13533382; doi:10.1371/journal.pgph.0007153)
Supplement: Supplementary file 1 [file pgph.0007153.s003.docx]

Inclusivity in global research

PLOS’ policy on inclusivity in global research aims to improve transparency in the reporting of research performed outside of researchers’ own country or community and ensures that PLOS publications reporting global research adhere to high standards for research ethics and authorship. Authors of relevant research articles may be asked to complete the questionnaire below, which outlines ethical, cultural, and scientific considerations specific to inclusivity in global research. This questionnaire may be requested when researchers have travelled to a different country to conduct research, if research uses samples collected in another country, research with Indigenous populations or their lands, or if research is on cultural artefacts. Researchers travelling to another country solely to use laboratory equipment will not normally be required to complete the questionnaire. However, the questionnaire can be requested at the journal’s discretion for any submission – if you have been requested to complete this questionnaire by the PLOS journal you submitted to, please do so.

Please complete the questionnaire below and include this as a Supporting Information file with your manuscript. Note that if your paper is accepted for publication, this checklist will be published with your article in the supporting information files. Please ensure that you reference the checklist in the main body of your manuscript. We suggest adding a subsection ‘Inclusivity in global research’ to your Methods section and adding the following sentence: “Additional information regarding the ethical, cultural, and scientific considerations specific to inclusivity in global research is included in the Supporting Information (SX Checklist)”

The questions have been designed to be applicable to a wide range of study types, and there are subsections for both human subjects research and non-human subjects research. If any of the questions are not relevant to your research please mark them as “N/A” as appropriate.

**Ethical considerations, permits and authorship**

*This section is applicable to all research types.*

Provide details as to who granted permissions and/or consent for the study to take place in the Methods section of your manuscript. This should include the names of **all** ethics boards, governmental organizations, community leaders or other bodies that provided approval for the study. If individuals provided approval refer to these people by their role or title but do not list their name(s).

Reported on page number: 6

If there were any deviations from the study protocol after approval was obtained please provide details of these changes in the Methods section of your manuscript.
Did this study involve local collaborators that are residents of the country where the research was conducted or members of the community studied? If you do not have any authors from said communities, please provide an explanation for this below.

Reported on page number:

Yes. The study was a collaborative effort involving Ethiopian professionals. It was designed to

be participatory and use a &quot;train-the-trainer&quot; approach with members of the local community. A local psychiatrist and a public health professional provided continuous supervision to guarantee

the program was carried out consistently and data collection was accurate. The research team

also included three authors from Addis Ababa, as well as a psychiatrist and mental health

professional who offered initial training for the intervention.

Through the inclusion of local experts in the adaptation process, the study highlighted cultural

importance. In order to ensure that the language and content were suitable for the local people,

Ethiopian and German mental health experts collaborated to adapt the original materials in a

workshop held in Addis Ababa. In addition to this Local school-aged children took part in

focused group discussions to review and adjust training materials, like posters and book marks,

to ensure they align with their cultural context and preferences. The intervention was delivered

by school staff (SN/HEWs). Two local young adults with mental disorder lived experience also

participated in the the contact-based workshop and shared the school children about their own

experience.

Everyone listed as an author should meet PLOS’ criteria for authorship and all individuals who meet these criteria should be included in the author byline, rather than the acknowledgements. For further information please see the journal’s Authorship Policy.

**Human subjects research (e.g. health research, medical research, cross-cultural psychology)**

Did you obtain written informed consent from a representative of the local community or region before the research took place? How did you establish who speaks for the community? Details of written informed consent obtained from study participants should be reported separately in the Methods section of your manuscript.

Yes, we initially obtained ethical approval and institutional permissions.

While assent was taken from the school children, the parents provided written informed consent

prior to actual data collection, the SN and HEWs also provided written informed consent.

The study used a participatory cultural adaptation strategy to build community leadership and

voice. It was carried out in cooperation with local mental health experts. The school children

were also invited to review and make changes to the intervention materials, making sure the

program represented the viewpoints of the community under study, better suit their cultural

background and preferences as young people.

How did members of the local community provide input on the aims of the research investigation, its methodology, and its anticipated outcome(s)?

Members of the local community provided significant input on the study’s aims, methodology and anticipated outcomes through a structured participatory cultural adaptation process. This collaboration was achieved through the following mechanisms.

Integrating local experts to provide their input on research methodology and implementation including adaption , translation and contexualization of the intervention materials (including public and mental health experts and moficiation of materials after having a focused group discussion with the schoolchildren and intervention delivery (by local SNs and HEWs and lived experience contribution)

When engaging with the local community, how did you ensure that the informed consent documents and other materials could be understood by local stakeholders?

To ensure that informed consent and other research materials were understandable and culturally appropriate for local stakeholders in Addis Ababa, all training materials and assessment scales were translated into Amharic, the local language. To ensure accuracy and conceptual consistency, the team used-back translation and had the materials reviewed by local professionals. A collaborative workshop was held in Addis Ababa, involving mental health experts from both Ethiopia and Germany. Through this workshop, the experts reviewed and adapted the materials. Local school aged youth were invited to review and modify the printed materials such as posters and bookmarks to ensure they align with their perspectives and preference. Finally, the intervention was delivered by local SNs and HEWs who were trained to provide additional explanations of mental health concepts during discussions to ensure students fully understood the information being presented.

Will the findings of the research be made available in an understandable format to stakeholders in the community where the study was conducted (e.g. via a presentation, summary report, copies of publications, etc.)? Please provide details of how this will be achieved.

The findings of this study will be disseminated to community stakeholders in a number of ways, mostly though the use of culturally appropriate materials created during the study and subsequently disseminated by academic institutions and local communities. These include: Publicy displayed materials: anti-stigma posters with culturally appropriate slogans (e.g., “I am not my mental illness”)these were put up in prominent places within schools, like libraries, to guarantee ongoing accessibility for the students. Additionally the school children received bookmarks with de-stigmatizing messages directly. In order to support the growth of mental health awareness and anti-stigmatization initiatives in schools, the study’s results will also be shared with important stakeholders who represent health, education, students and others.

**Non-human subjects research using specimens/ animals collected as part of the study, or those housed in archival collections. Examples include archaeology, paleontology, botany and zoology.**

Did the permission you obtained from a local authority to perform the study include an agreement on access to outputs and benefit sharing? This may include procedures to enable fair distribution of the benefits and resources arising from the research performed. Please include any details of Prior Informed Consent and Benefit Sharing Agreements obtained. These may be required by field-specific regulations, for example the Convention on Biological Diversity (CBD) and the associated Nagoya Protocol.

If the material used in your study was imported, please A) provide the year it was imported and B) indicate whether permits were obtained to import/export the materials used, C) provide details of any permits obtained. If this information is not available, please indicate this.

If you used archival specimens, please state how the material used in your study was acquired by the institute it is held in and provide details of any permits obtained for the original excavations/ sample collection. If this information is not available, please indicate this.

How was the potential cultural significance of the materials collected in your study to local communities considered in your research design? Were Indigenous peoples and/or local researchers and institutions involved with archaeological excavations / collection of specimens? If so, please provide a description of their involvement.

If your manuscript includes photographs of human remains please indicate whether authors obtained permission from descendants or affiliated cultural communities to do so.
