# Supplementary material for: From stigma to support: Assessing the impact of school mental health literacy program in Addis Ababa, Ethiopia
Source: PLOS Glob Public Health. 2026 Sep 1;6(9):e0007153. doi: 10.1371/journal.pgph.0007153 (PMC13533382; doi:10.1371/journal.pgph.0007153)
Supplement: Supplementary file 2 [file pgph.0007153.s005.docx]

Response to Reviewers

1. Please include a complete copy of PLOS’ questionnaire on inclusivity in global research in your revised manuscript. Our policy for research in this area aims to improve transparency in the reporting of research performed outside of researchers’ own country or community. The policy applies to researchers who have travelled to a different country to conduct research, research with Indigenous populations or their lands, and research on cultural artefacts. The questionnaire can also be requested at the journal’s discretion for any other submissions, even if these conditions are not met. Please find more information on the policy and a link to download a blank copy of the questionnaire here: <https://journals.plos.org/globalpublichealth/s/best-practices-in-research-reporting>. Please upload a completed version of your questionnaire as Supporting Information when you resubmit your manuscript.

Surafel has completed this and I will upload the questionnaire under supporting information.

*We have filled the questionnaire on inclusivity in global research and uploaded it to the portal.*

2. Please amend your detailed Financial Disclosure statement. This is published with the article. It must therefore be completed in full sentences and contain the exact wording you wish to be published.

i. Please clarify all sources of financial support for your study. List the grants, grant numbers, and organizations that funded your study, including funding received from your institution. Please note that suppliers of material support, including research materials, should be recognized in the Acknowledgements section rather than in the Financial Disclosure. 
ii. State the initials, alongside each funding source, of each author to receive each grant. For example: "This work was supported by the National Institutes of Health (####### to AM; ###### to CJ) and the National Science Foundation (###### to AM)."
iii. State what role the funders took in the study. If the funders had no role in your study, please state: “The funders had no role in study design, data collection and analysis, decision to publish, or preparation of the manuscript.”
iv. If any authors received a salary from any of your funders, please state which authors and which funders.

*We have added this financial disclosure statement. The study was supported by a research grant (grant to E.G.) received through the network funds projects of the Center for International Health (CIH^LMU^) at the Hospital of Ludwig-Maximillian’s-University Munich. The CIH^LMU^ receives funding from the German Developmental Exchange Service (DAAD) through the program “exceed – excellence Centers for Exchange and Development”, support by the Federal Ministry of Economic Cooperation and Development (BMZ). We have added this financial disclosure statement.*

*The funders had no role in study design, data collection and analysis, decision to publish, or preparation of the manuscript.*

3. Please ensure that your Ethics Statement is available in its entirety at the beginning of your Methods section, under a subheading 'Ethics Statement'.

*We have moved the ethics statement to the beginning of the methods section under the subheading ‘Ethics Statement’ (lines 159-165) as suggested.*

4. Please upload separate figure files in .tif or .eps format. Also, remove the figures from your manuscript file but keep the legends.

For more information about figure files please see our guidelines:

<https://journals.plos.org/globalpublichealth/s/figures> 
<https://journals.plos.org/globalpublichealth/s/figures#loc-file-requirements>

*We have removed the figures and kept the legends and uploaded it as a tif and uploaded as figure 1 and 2.*

5. We have noticed that you have cited Tables 3 and 5 in the manuscript file but there are no corresponding tables in the manuscript. Please amend your manuscript to include this table, noting that tables should not be uploaded as individual files.

*We noticed the mistake and realized there was an error made during the editing process in regards to the tables, the citations have been updated in correct correspondence to the appropriate table as mentioned in the text.*

6. We have noticed that you have cited ‘supplement 1 and 2’ in your manuscript. However, there are no corresponding files uploaded to the submission. Please upload them as separate files with the item type 'Supporting Information'.

*We have removed supplement 1, due to not receiving copyright from the authors. We have thus update the manuscript to reflect only supplement 2 becoming supplement 1.*

7. In this instance it seems there may be acceptable restrictions in place that prevent the public sharing of your minimal data. However, in line with our goal of ensuring long-term data availability to all interested researchers, PLOS’ Data Policy states that authors cannot be the sole named individuals responsible for ensuring data access (<http://journals.plos.org/plosone/s/data-availability#loc-acceptable-data-sharing-methods>).

 Data requests to a non-author institutional point of contact, such as a data access or ethics committee, helps guarantee long term stability and availability of data. Providing interested researchers with a durable point of contact ensures data will be accessible even if an author changes email addresses, institutions, or becomes unavailable to answer requests.
 
 Before we proceed with your manuscript, please also provide non-author contact information (phone/email/hyperlink) for a data access committee, ethics committee, or other institutional body to which data requests may be sent. If no institutional body is available to respond to requests for your minimal data, please consider if there any institutional representatives who did not collaborate in the study, and are not listed as authors on the manuscript, who would be able to hold the data and respond to external requests for data access? If so, please provide their contact information (i.e., email address). Please also provide details on how you will ensure persistent or long-term data storage and availability.

If the reviewer comments include a recommendation to cite specific previously published works, please review and evaluate these publications to determine whether they are relevant and should be cited. There is no requirement to cite these works unless the editor has indicated otherwise.

*We have provided the information regarding the data from this study can be accessed from: Saint Paul’s Hospital Millennium Medical Colleges Institutional Research Ethics Review Committee. Email: irb@sphmmc.edu.et*

Response to Reviewer #1 Feedback:

• If I understand correctly, the study design used is “Pre-Post quasi experimental without control design”. But in the methods section it just says pre-post quasi experimental design. Hence, it is better to provide the complete design name.

*We* *agree and have updated the correct name of the study design in the manuscript to Pre-Post quasi experimental without control design under Lines 167-168*

• Providing a flow-chart of methods will be helpful

*We will provide a flow chart of methods as figure 1 and uploaded as figure 1.*

• Making the flow of intervention more precise is very critical. It can also be provided as a supplementary material.

*This question is addressed with our response to the previous question. In our flow chart we show the flow of the intervention and how the SN/HEWs were trained, we have uploaded as supplement 3.*

• Need to mention how many SN & HEWs were trained and a bit more background of those personnel.

*We agree and we added this description to the updated manuscript with the number of SN and HEWs which took part in delivering the intervention and their background information (lines 223-229) . “All selected staff had at minimum one year of working experience, resulting in a total of seven SNs/HEWs who were trained to implement the anti-stigma intervention at the schools. There was a total of (n=7) SN and HEW whom had an average age of 37.29 years (SD=11.22), and an average of 8.14 years of working experience (SD=7.56), 7 of the staff had a Bachelor of Science and 3 had a Masters of Science.”*

• Contents of the interventions provided through PowerPoint presentations/talks are inadequate, it is important for the readers to know what topics are covered in those talks.

*We agree and have added in more information regarding the PowerPoints in the intervention subsection in the methods section (lines 307-311). “Classroom training sessions delivered by SNs/HEWs included PowerPoint lectures on varying mental health topics such as stigma and discrimination, common mental disorders: anxiety, depression, psychosis, and attention deficit hyperactivity disorder, with their etiology and their manifestations. Students were also shown options for treatment, and recommendations for better mental health. They were also given information of the available mental health providers in the country.”*

• In the discussion, it will be more appropriate to briefly reflect on the challenges of such interventions based in schools and focusing on mental health stigma reduction.

*We agree and we have expanded on this in the discussion section and have added the challenges we personally faced during the intervention (lines 642-646) “Challenges encountered in implementing this type of intervention in a school-based setting stemmed from deeply rooted cultural stigma, low levels of mental health awareness and the widespread belief that mental illness is attributed due to supernatural causes. These beliefs are embedded within the Ethiopian cultural and societal perspectives and became evident as the intervention was taking place.”*
