# Supplementary material for: From stigma to support: Assessing the impact of school mental health literacy program in Addis Ababa, Ethiopia
Source: PLOS Glob Public Health. 2026 Sep 1;6(9):e0007153. doi: 10.1371/journal.pgph.0007153 (PMC13533382; doi:10.1371/journal.pgph.0007153)
Supplement: Supplementary file 3 [file pgph.0007153.s006.docx]

Response to Reviewers:

Reviewer #2: Suggestions for modification and questions to clarify some statements are provided as follows.

1. The abbreviation should be mentioned for the first time. For example: SNs and HEWs in line 167 instead of line 177, and SWM in line 180. x

*We agree and we have modified the explanation earlier in line 164.*

*Thank you for this observation. We have thoroughly reviewed the manuscript and ensured that all abbreviations including School Nurses (SNs), Health Extension Workers (HEWs), and author initials (SWM) are defined at their very first appearance in the text.*

2. Authors should explain how the planned sample is selected (line 250). x

*We agree and we have gone into a further detailed explanation regarding our sampling process in line 209 to 217: “To enroll the study participants, a list of schools was obtained from the Addis Ababa Health Bureau. From this list, four schools (two private and two public) were selected based on two criteria: first* *the school’s willingness to conduct the study and second, location due to proximity with the School of Public Health, which facilitated and supervised the study with the schools. After that, the sample size was then distributed proportionally between public and private schools according to the number of students in each school. Subsequently, sections were chosen in a lottery method from each selected school. Finally, a list of students from the selected sections was prepared and participants were recruited.”*

*We have expanded the participants subsection to clarify the sampling strategy. A multi-stage sampling approach was used:*

1. *Sub city and school selection: Four high schools (two public and two private) across Addis Ababa were purposefully selected based on* *the school’s willingness to conduct the study and second, location due to proximity with the School of Public Health, which facilitated and supervised the study with the schools and diversity in their administrative structure and socioeconomic catchments.*
2. *Section Selection: Within each school, 9^th^-grade sections (classrooms) were randomly selected using a lottery method.*
3. *Student selection: All eligible students within the selected sections whose parents provided informed consent and who gave personal assent were invited to participate, targeting approximately 50 students per school to reach the planned sample.*

3. No text describes how students' mental health status was assessed. The detailed inclusion and exclusion criteria should be included.

*We appreciate to clarify, regarding mental health status, no screening or assessment of mental health disorders was conducted as part of eligibility. This is because the intervention was designed as a universal school-based mental health literacy and anti-stigma programme, delivered to all eligible students irrespective of risk status. In line with established prevention frameworks, universal interventions are implemented without prior identification of mental health conditions (Institute of Medicine, 1994).*

*This study evaluated a universal school-based public health intervention targeted at the general student population rather than a clinical trial targeting specific psychiatric conditions. Therefore, individual clinical mental health status was not formally assessed or used as an eligibility criterion.*

*We have explicitly added the inclusion and exclusion criteria to the participant’s section in line 205-209.*

*Inclusion criteria: Being a regularly enrolled 9^th^ grade student at one of the four selected schools during the 2022/2023 academic year, providing a written informed assent and, obtaining signed parental informed consent.*

*Exclusion criteria: Sevier acute physical or cognitive illness on the days of data collection that precluded completion of the self-administered digital questionnaire.*

*The selection of Grade 9 students was guided by both developmental and practical considerations. From a developmental perspective, adolescence is a key period for the onset of mental health disorders, making this age group particularly relevant for preventive mental health literacy interventions (Kessler et al., 2005; Ozer et al., 2022). From a contextual and implementation perspective within the Ethiopian education system, Grade 8 students were excluded because they are engaged in preparation for the national Grade 8 exit examination administered by the Ministry of Education, which represents a high-stakes academic transition. Including Grade 8 students during this examination period would likely have introduced substantial academic burden and could have interfered with both examination preparation and intervention delivery. Therefore, Grade 9 students were selected as a more appropriate and stable cohort for implementing the intervention across both public and private schools.*

4. In line 219, it is mentioned that a total of 7 SNs and HEWs, but there was a total of 10 staff according to their education level. Therefore, it is necessary to clarify.

*We apologize for the typographical discrepancy in the text which caused confusion. A total of seven (7) health staff members were recruited and trained across the four schools (one school utilizing a single health worker who managed the assigned cohort due to scheduling layout, lines 251-253.*

*The sentence detailing education levels contained an arithmetic error in the breakdown. It has been corrected to read: “A total of 7 SN/HEW staff implemented the intervention. Among them, 5 held a Bachelor of Science (B.Sc.) degree and 2 held a Master of Science (M.Sc.) degree”. We have cross-checked this with our records and updated the text and demographic narratives accordingly, lines 292-293.*

5. Subtitles should be written perfectly instead of one word like “Measures” in line 230.

*We agree and we have updated the subtitles to reflect that in line 261. In accordance with the reviewer’s suggestion and standard journal formatting, we have revised the single-word subheadings to be more descriptive. For example, “Measures” has been changed to “Data Collection Instruments and Outcome Measures” and “Intervention” to “Intervention Structure and Components”*

6. Because the Cronbach alpha values are not so good (0.63), is there any removal of original items for the local study before data collection? ( line 239 and line 246)

*During the translation and cultural adaptation phase, the expert panel chose to retain all original scale items to preserve the structural validity established by Link et al. (2020), rather than removing items prior to data collection. We acknowledge that Cronbach alpha values of 0.63 (for the knowledge and Positive Attitude Scale and Stigma Awareness and Action Scale) are modest. Due to multi-dimensional nature of broad constructs such as “stigma awareness”, these lower internal consistencies are common when established western psychological instruments are translated and administered to cross-cultural adolescents in a LMICs.*

*In the revised manuscript, we expand on this in the Limitations section line (720-730), noting that while the scales remained sensitive enough to capture highly significant pre-post shifts, future scaling up would benefit from full psychometric validation and potential optimization of item wording for Amharic-speaking adolescents.*

7. Interactive activities “writing a letter to a person with lived experience” are not clear (line 278), and so easier-to-understand examples should be provided. In this example, what would the author like to say to a person with lived experience? During this activity, how does the investigator deal with the ethical issues, especially regarding confidentiality and the rights of the participants? These issues should be described.

*We appreciate the opportunity to elaborate on this component of the intervention.*

*During the classroom training, students were asked to write an anonymous, brief letter expressing their thoughts, support, or questions to a hypothetical peer or individual experiencing a mental health struggle. The primary educational objective was to foster empathy and perspective-taking. Examples of what students typically wrote included messages of solidarity (e.g., “I want you to know that you are not alone or having a mental illness does not change your value”) or a newly gained awareness (e.g., I used to think that people could just choose to stop being depressed, but now I understand it’s a medical condition and I want to support you”), lines 391-398.*

*Regarding the ethical and confidentiality measures:*

*To ensure participants confidentiality, the following steps were strictly enforced:*

1. *Anonymity: Students were explicitly instructed not to write their names, section numbers, or any identifying markers on the letters.*
2. *Voluntary participation: Students were informed that writing the letter was a reflective learning exercise, and they could choose not to submit their letters to the facilitators if they felt uncomfortable.*
3. *Data handling: The letters were collected, and reviewed by only the facilitators and they were not shared publicly or linked to the quantitative ODK survey data. Finally, they were securely discarded after the project completion.*

*We have integrated these structural details into the manuscript’s intervention description lines 333-347..*

**8. Also, what does young adults mean in line 279? How did the author find out about such people who are living with a mental disorder? What is the sampling procedure?**

*By “young adults,” we refer to individuals aged 20-25 years. This age group was purposefully selected so that the contact-based session featured individuals close in generation to our adolescent participants, making their lived experience more relatable, line 313.*

*Recruitment and Sampling Procedure:*

*The two individuals with lived experience were recruited via purposive convenience sampling through clinical networks at tertiary psychiatric unit in Addis Ababa. They were clinically stable, well-functioning, undergoing regular follow-up, and deemed emotionally and psychologically well enough by their treating psychiatrists to participate in public speaking.*

*Their participation was completely voluntary. Formal written consent was obtained from both. They received detailed briefing on the school environment, and main areas they would discuss by SWM. They were compensated for their time and transportation. We have added these details to the manuscript to maximize transparency lines 236-243.*

9. Explanation about posters is included twice, with a slogan “I am not my mental illness,” in lines 300 and 313. So these sentences should be modified.

*Thank you for noticing this redundant wording. We have consolidated the descriptions of the printed materials into a single paragraph in the revised manuscript and removed the repetitive mentions.*

**10. How was the sample size determined for this study?**

*The sample size was determined primarily by practical feasibility, resource and logistical availability. We targeted a cluster layout of 50 students per school across the 4 school with distinct institutional environments (targeting 200 students in total) to ensure a sufficient sample for paired non-parametric and parametric analyses while maintaining operational oversight and operational fidelity. We have updated the manuscript to reflect this update, lines 211-217.*

**11. Is there any loss to follow up after the intervention?**

*We thank the reviewer for this important question regarding participant retention. Of the 200 students enrolled at baseline, 186 (93%) completed both the pre- and post-intervention assessments. The 14 students (7%) who did not complete the post-intervention questionnaire were absent on the day of post-intervention data collection (three weeks after the intervention). No students withdrew due to unwillingness to participate, and no students declined enrolment at baseline after being approached. No systematic differences in baseline sociodemographic characteristics were identified between completers and non-completers based on available data. No imputation or replacement of missing data was conducted; the analysis was therefore performed on the complete-case sample of 186 participants.*

*Despite collecting a baseline data from 200 students across the four schools, at the 3-week post-intervention evaluation, 186 students successfully completed the follow-up assessment, representing a loss to follow-up 7% (n=14). The loss to the follow-up resulted from students’ absenteeism on the day of the post assessment or incomplete entry profiles on the ODK application. This can be observed from the Participants Flow Diagram, lines 222-223.*

12. In line 318, the title should be “Cultural adaptation steps for intervention package development” rather than Cultural Adaptation.

*We agree with this comment and have modified according to the suggestion in line 370.*

13. In line 342, the authors mention Spearman's Rank Correlation, but in the results section, no description is found for this analysis.

*We appreciate this observation. During our analytical stages, Spearman’s rank correlations were run as exploratory checks to examine bivariate relationships prior to building the linear regression models. Because the key predictive findings are fully captured within multiple linear regression output (Table 3), the redundant bivariate Spearman text was removed from the Statistical Analysis section to ensure alignment with the reported results.*

14. A working definition of “school affiliation” should be included. (line 408*)*

*We agree and we have added an operational definition of school affiliation in line 442-446.* *school affiliation is operationalized as a categorical dummy-coded covariate indicating which of the four participating high schools (school 1, school 2, school 3, or school 4) a student was enrolled in. This account controls for structural, environmental, and socio-demographic variations across private and public-school settings.*

**15. Is there any multicollinearity check before linear regression, because independent variables may be linked to each other, like the knowledge and attitude scale with knowledge and attitude change, and the school affiliation of different schools? (for Table 3)**

*We thank the reviewer for raising this important point. We conducted a multicollinearity check (variance inflation factors) for the regression model reported in Table 3. All VIF values were low (adjusted GVIF range: 1.02–1.23), well below conventional thresholds of concern (e.g., VIF > 5), indicating that multicollinearity among baseline social distance, baseline knowledge and attitudes, and school did not pose a problem for the interpretation of the regression coefficients. Tolerance values exceeded 0.20, indicating that multicollinearity was not a concern. We have added this information to the Methods section, lines 541-544.*

16. In line 566, there is a discussion point regarding education level referencing Girma et al., 2013. In the current study, there is no consideration of the education level as a predictor variable in the analysis phase, and so this discussion is not aligned with the findings.

*We agree with the reviewer that individual student education level was homogeneous (all participant were in the 9^th^ grade) and not evaluated as analytical predictor. Girma et al., 2013 was referenced to provide general societal context regarding how mental health stigma is perceived and experienced in the broader Ethiopian population. However, to maintain close alignment with our findings, we re-framed this paragraph to focus strictly on how school environments and youth-targeted educational exposures influence stigma and we removed the non-aligned aspect on parent/child educational levels.*

17. A label should be included for the study flow diagram.

*We agree and we have added a label for the study flow diagram.*

18. Line spacing and alignments should be modified to meet the Journal format.

*We have reformatted the entire manuscript, including line spacing, margins and section headings to conform fully with the journal’s style guidelines.*

Reviewer #3: Thank you for the opportunity to review this manuscript. The topic is highly relevant to global public health, particularly in low- and middle-income countries where adolescent mental health services and stigma reduction programs remain limited. The manuscript addresses an important gap by evaluating a culturally adapted school-based mental health literacy intervention in Ethiopia. I particularly appreciate the authors’ efforts to culturally adapt the intervention and involve local stakeholders and adolescents in the process. The paper is generally well organized, and the findings are encouraging.

At the same time, there are several methodological and reporting issues that should be clarified before the manuscript is suitable for publication.

**1. Study design and interpretation of findings
The main limitation of the study is the absence of a control group. Since this is a pre-post quasi-experimental study without comparison participants, it is difficult to determine how much of the observed improvement can truly be attributed to the intervention itself. Some of the wording in the Results and Discussion sections implies causality or effectiveness more strongly than the design can support.
I would encourage the authors to moderate some of the conclusions and frame the findings more cautiously, for example by stating that the intervention was “associated with improvements” rather than definitively “demonstrated effectiveness.”
The limitation is acknowledged later in the manuscript, but it should be emphasized more clearly when interpreting the results.**

*We* *agree with this foundational critique. Given the pre-post quasi-experimental design without a parallel control group, we cannot definitively rule out other factors contributing to the observed post intervention improvements.*

*We have reviewed the entire manuscript including the Abstract, Results, Discussion and Conclusion sections to remove causal language. Strong terms such as “demonstrated effectiveness” or “proven impact” have been systematically replaced with cautions, association-based terminology, such as “was associated with significant improvements” and “showed preliminary promise”. We have also expanded our discussion of this limitation.*

**2. More detail is needed regarding participant recruitment and follow-up
The manuscript would benefit from clearer reporting of participant flow.
The authors mention that 200 students were targeted, while the final analyses included 186 participants. It would help readers to know:
- how many students declined participation,
- whether there was any loss to follow-up,
- whether missing data occurred,
- and whether all students completed both pre- and post-intervention assessments.
A simple participant flow diagram would improve transparency considerably.** **In addition, more explanation regarding how the four schools were selected would strengthen the manuscript.**

*To maximize transparency, we have submitted a comprehensive Participant Flow Diagram (Figure 1) tracking the progress of the cohort. Specifically:*

- *Initial Target: 200 students across 4 schools.*
- *Exclusions/ Declined: 0 Parents/students actively declined but 14 individual student data files were excluded from final analysis due to absence during the 3-week post-test data collection day or incomplete electronic survey forms.*
- *Final Analysis Cohort: 186 students who completed both the baseline and post-intervention assessment in full.*

*Additionally, we have expanded the methods text to explain our purposive selection of the four schools to capture structural diversity (two public and two private schools) across different sub cities in Addis Ababa, lines 246-262.*

**3. Psychometric properties of the scales
Several scales showed relatively low Cronbach alpha values, particularly around 0.63–0.67. While this may be understandable in cross-cultural adolescent research, the implications should be discussed more carefully.
Since cultural adaptation is a major strength of this study, it would be helpful to provide more information on:
- whether pilot testing was conducted,
- whether adolescents had difficulty understanding any items,
- and whether the translated instruments were formally validated in the Ethiopian context.**

*We thank the reviewer for highlighting this important point. The tools were translated into Amharic and back-translated, followed by a qualitative content validation via our youth focus groups during the adaptation phase. However, a formal psychometric validation study of these scales within the Ethiopian context has not been published yet.*

*The moderate alpha values (0.63-0.67) reflect the performance of these translated western validated items among Ethiopian adolescents for the first time. We have explicitly described this in our Limitations section, addressing these psychometric properties, noting that while the scales exhibited a good sensitivity to successfully detect the shifts, certain reverse-coded or abstract stigma concepts may require further localized modification and validation in future scale-up designs.*

*Some of the study measures demonstrated moderate internal consistency in the present sample, with Cronbach's alpha values ranging from 0.63 to 0.67 for several scales. This may reflect the use of binary response formats for some instruments, which typically yield lower reliability estimates than Likert-type scales, as well as the multidimensional nature of stigma-related constructs, encompassing distinct but related domains such as knowledge, attitudes, avoidance, and social distance. Therefore, the findings should be interpreted with appropriate caution.*

**4. Statistical analysis needs clarification
The statistical approach is generally appropriate, but several parts need clearer explanation.
First, the rationale for using Wilcoxon signed-rank tests for the binary-response scales should be explained more explicitly.
Second, the regression model predicting post-intervention social distance would benefit from further clarification. Including both baseline scores and change scores in the same model may introduce statistical dependency. The authors should explain why this approach was chosen and whether multicollinearity was assessed.**

*We thank the reviewer for these important points and address each in turn.*

***First,*** *for the Stigma Awareness and Action Scale, the Avoidance and Discomfort Scale, and the Personal Help-Seeking Scale, individual binary items were summed to create participant-level total scores. Because these summed scores are ordinal, bounded, and not normally distributed, pre- and post-intervention differences were analyzed using the Wilcoxon signed-rank test, which is appropriate for paired, non-normally distributed data of this kind. We have clarified this rationale in the Methods section.*

***Second (regression model),*** *we appreciate the reviewer raising this concern. Upon reflection, we agree that including both baseline knowledge and attitudes and the change in knowledge and attitudes in the same model predicting post-intervention social distance introduced statistical dependency between predictors, since the change score is mathematically derived from the same baseline value already included in the model. To address this, we revised the regression model to instead include baseline social distance, baseline knowledge and attitudes, and school affiliation as predictors of post-intervention social distance, removing the change score to avoid this redundancy. This revised model also directly addresses the comment regarding the omission of baseline outcome levels as a covariate. We additionally assessed multicollinearity among the revised model's predictors using variance inflation factors (VIFs) and tolerance values; all VIFs were low (adjusted GVIF range: 1.02–1.23) and all tolerance values exceeded conventional thresholds (range: 0.66–0.96), indicating no problematic multicollinearity. The updated model, results, and multicollinearity diagnostics are now reported in the revised Table 3 and accompanying Methods and Results.*

**5. 5. Intervention implementation and fidelity
The intervention appears thoughtfully designed, especially the combination of classroom teaching, contact-based sessions, and printed materials. However, more detail is needed regarding implementation consistency across schools.
For example:
- Were all sessions delivered in the same way?
- Was fidelity formally monitored?
- Were trainers observed or supervised using a checklist?
- Did the researchers assess variability between schools or trainers?
Given that multiple nurses and health extension workers delivered the program,** this information is important.

*We appreciate this important focus on implementation science. To guarantee the intervention uniformity across the four school, we implemented the following quality controls:*

- *Standardization: All seven facilitators underwent an identical, centralized 2-day training led by the local mental health professionals (SWM and YG, an M.SC. clinician).*
- *Fidelity monitoring: Core research team members (SWM and FAW) conducted daily field supervision visits across the four school sites. Facilitators utilized standardized PowerPoint slide sets and a unified session timeline. In addition to this, we used similar printed materials and similar individuals with lived experience for the contact-based sessions.*
- *School variability: School affiliation was integrated directly as a covariate in our linear regression model (Table 3).All school nurses and health extension workers received identical two-day standardized training delivered by the same two mental health professionals. Standardized PowerPoint slides were provided to ensure content consistency across all four schools. During the intervention week, ongoing supervision was provided by the supervising psychiatrist (SWM) and a public health professional (FAW), who made site visits to each school and observed the session per school. While a formal structured fidelity checklist was not used - which we acknowledge as a limitation - no substantial deviations from the intervention protocol were identified during supervision visits. We further note that school affiliation was tested as a predictor in the regression model and was not found to be a significant factor, providing indirect evidence of sufficient cross-school delivery consistency. This information has been added to the Intervention section of the revised manuscript, and the absence of a formal fidelity tool has been added to the Limitations section.*

**6. Social desirability bias should be discussed more fully
Most outcomes relied on self-reported attitudes and intentions, which may be influenced by social desirability bias, especially shortly after a mental health awareness intervention.
This is particularly relevant because:
- students were aware of the study objectives,
- the intervention occurred within schools,
- and post-testing was conducted only three weeks later.
I recommend expanding this point in the limitations section.**

*This is an excellent point. Because students were fully aware of the anti-stigma objectives and completed evaluations within their respective schools just three weeks after interacting with facilitators and individuals with lived experience, their post-test response may be affected by social desirability bias. We have included this in our limitation section to thoroughly discuss how this potential bias might lead to over-reported help-seeking intentions or idealized attitude selections, underscoring the need for objective behavior measures and long-term, unannounced follow-ups in future studies.*

7. Language and grammar
The manuscript is generally understandable, but there are several grammatical and typographical issues throughout the paper that should be corrected during revision. Examples include:
- “one in meets the criteria”
- “0.04 psychiatrists per 100,000 peoples”
- “theses scores” instead of “these scores”
A careful language edit would improve readability.

*We agree and have gone through the manuscript in detail to improve readability.*

8. Strength of the manuscript
One of the strongest aspects of this work is the attention given to cultural adaptation and youth participation. This adds substantial value to the study and makes the intervention more relevant for LMIC settings. I encourage the authors to emphasize this practical contribution further in the Discussion.

**Are there any other background information or thoughts regarding the process of the cultural adaptation, behind the scenes.**

*We agree and we have gone into depth more regarding our approach.*

*We are grateful for the reviewer’s positive reinforcement of our cultural adaptation and Patient and Public Involvement (PPI) framework. In response, we expanded our Discussion section to highlight the real-world utility of using frameworks like the 10-step mhCACI and co-designed anti-stigma materials with local youth focus groups. We emphasized that this participatory model ensures contextual safety and relevance, and can serve as a replicable blueprint for scaling public health interventions across other LMIC settings.*

**Reviewer #4: Overall Assessment**
This manuscript addresses an important issue in global mental health, namely the reduction of mental health stigma among adolescents in low- and middle-income countries through school-based mental health literacy interventions. The topic is timely and relevant, particularly given the limited mental health resources and low mental health literacy reported in many LMIC settings. The cultural adaptation of the intervention for the Ethiopian context and the inclusion of a contact-based component are notable strengths.
The reported improvements in mental health knowledge, attitudes toward individuals with mental illness, social distance, and help-seeking intentions are encouraging and broadly consistent with previous research on stigma-reduction interventions among adolescents.
However, several substantial methodological and reporting limitations limit the interpretability of the findings. In its current form, the manuscript does not yet provide sufficiently rigorous evidence to support the conclusions presented. Major revisions would be required to address the issues outlined below.

**Major Comments
1. Study design and causal interpretation
The study uses a pre–post design without a comparison or control group. While such designs may provide preliminary evidence regarding potential intervention effects, they do not allow causal attribution of observed changes to the intervention itself.
Several alternative explanations remain possible, including:
• temporal or maturation effects
• social desirability bias
• exposure to external influences during the study period
Although the manuscript reports statistically significant improvements across several outcomes, these findings should be interpreted as associations observed following the intervention, rather than evidence of intervention effectiveness.
The Discussion and Conclusion sections should be revised to avoid causal language and more explicitly acknowledge the limitations of the study design.**

*We agree with this foundational critique. Given the pre-post quasi-experimental design without a parallel control group, we cannot definitively rule out other factors such as temporal or maturation effects, social desirability bias and exposure to external influences during the study period contributing to the observed post intervention improvements.*

*We have reviewed the entire manuscript including the Abstract, Results, Discussion and Conclusion sections to remove causal language. Strong terms such as “demonstrated effectiveness” or “proven impact” have been systematically replaced with cautions, association-based terminology, such as “was associated with significant improvements” and “showed preliminary promise”. We have also expanded our discussion of this limitation.*


**2. Short follow-up period
The post-intervention assessment was conducted three weeks after the intervention, which represents a relatively short follow-up period for evaluating changes in attitudes and stigma-related behaviors.
It is therefore unclear whether the observed improvements in knowledge, attitudes, social distance, and help-seeking intentions are sustained over time. The authors should discuss this limitation more explicitly and suggest that future studies incorporate longer follow-up periods to evaluate the durability of intervention effects.**

*We completely agree. A three- week evaluation window only captures immediate, short-term changes and cannot confirm long-term attitudinal adjustments. We have revised our Limitations section to emphasize this and we explicitly state that future studies should aim for a longer assessment period to measure the durability and decay rates of these stigma-reduction gains.*

**3. Statistical reporting and analytical transparency
The statistical reporting would benefit from additional detail to improve methodological transparency. In particular, the manuscript should consider including:
• confidence intervals for key estimates
• information on the reliability or internal consistency of the measurement scales used (e.g., Cronbach’s alpha)
• clarification of whether clustering at the school level was considered in the analysis
Given that participants were recruited from schools, potential intra-cluster correlation may influence variance estimates and should be addressed or acknowledged.**

*We agree with the reviewer’s comment. We improved our statistical reporting with the following measures:*

- *Confidence Intervals: we have added 95% confidence intervals for all primary parametric t-test estimates across text and tables.*
- *Scale Reliability: Cronbach’s alpha values have been clearly emphasized within the Data Collection Instruments and Outcome Measures section.*
- *School-Level Clustering: To account for cluster effects across the four schools, school affiliation was included as a fixed-effect categorical covariate in our linear regression model (Table 3). The analysis confirmed that school affiliation was not a significant predictor of social distance variance, suggesting minimal intra-cluster bias across the schools*

**4. Data availability and journal policy compliance
The Data Availability Statement indicates that the data are available only upon reasonable request due to ethical restrictions. However, journals such as PLOS typically require that all underlying data supporting the results be publicly available, unless there are clear ethical or legal constraints.
The authors should clarify whether a de-identified dataset could be deposited in a public repository or provided as supplementary material in accordance with journal data-sharing requirements.**

*We fully support the journal’s commitment to open science and data reproducibility. In compliance with data sharing policies, we have prepared a fully anonymized, de-identified dataset. This dataset will be uploaded as a Supplementary Information File (Dataset S1) during resubmission of our revised version.*

5. Intervention description and reproducibility
The manuscript would benefit from a more detailed description of the intervention and its cultural adaptation process.
Specifically, the authors should provide additional information on:
• how the intervention was culturally adapted for the Ethiopian context
• the training and qualifications of facilitators
• the duration and structure of each intervention component
Providing these details would improve the reproducibility and external applicability of the intervention for other LMIC settings.

*We agree that detailed description of the intervention and its cultural adaptation process will maximize its reproducibility. We have expanded our Intervention Structure and Components and Cultural Adaptation subsections to include the following details:*

- *Facilitator Qualification: we described that the 7 facilitators comprised 4 private SNs and 3 government HEWs, all with a minimum of 1 year of school health experience, and holding either B.Sc. or M.Sc. in nursing or public health.*
- *Training Intensity: we specify that facilitators underwent a mandatory 2-day intensive training workshop covering adolescent mental health, anti-stigma strategies, crisis mitigation protocol and digital data collection tracking via ODK.*
- *Component Structure: we provide a breakdown of specific contents and time frames of the 30-50 minute curriculum slots over the 4 active days, detailing the operational timeline for the lectures, letter writing and contact based sessions and the placement of anti-stigma printed materials***.**

6. Concerns Regarding Interpretation of Results
Overinterpretation in the Conclusion
The current conclusion appears to overstate the strength of the evidence relative to the study design.
Example (Lines 656–658)
“This study demonstrated successful reduction in stigma toward individuals with mental health problems and improvements in mental health knowledge and help-seeking behavior…”
This statement implies demonstrated effectiveness, which is not supported by the current study design. A more appropriate interpretation would be that improvements were observed following the intervention, rather than that the intervention was proven to be effective.

Example (Lines 659–661)
“Such interventions can enhance mental health knowledge, reduce stigma and potentially improve …”
This sentence appears to generalize the findings beyond the study population. Conclusions should remain closely aligned with the evidence generated from the current study.

*We accept this criticism. The specific phrases flagged by the reviewer have been rewritten to reflect proper interpretation of our findings.*

- *The first example has been revised to: “ The study observed significant short-term improvements in adolescent mental health literacy, help-seeking intentions, and reduction in expressed social distance following a culturally adopted school-based program in Addis Ababa Ethiopia.”*
- *The second sentence has been revised to: “Within our study sample, this multi-component approach was associated with improved literacy and reduced stigmatizing attitudes, suggesting that similar school-based models warrant further evaluation in LMICs.”*

7. Introduction: Clarification of the Knowledge Gap
While the Introduction provides relevant background on mental health stigma and adolescent mental health literacy in LMICs, the specific research gap remains insufficiently articulated.
The authors should more clearly:
• synthesize existing evidence on school-based anti-stigma interventions in LMICs, and
• explicitly state what gap in the literature this study aims to address.
Clarifying this point would strengthen the conceptual positioning of the study.

*We appreciate this valuable recommendation to strengthen our studies’ conceptual framework. We have revised the final section of our introduction to include a more thorough synthesis of school-based interventions and demonstrate the existing gaps in LMICs.*
