# Supplementary material for: From stigma to support: Assessing the impact of school mental health literacy program in Addis Ababa, Ethiopia
Source: PLOS Glob Public Health. 2026 Sep 1;6(9):e0007153. doi: 10.1371/journal.pgph.0007153 (PMC13533382; doi:10.1371/journal.pgph.0007153)
Supplement: Supplementary file 4 [file pgph.0007153.s007.docx]

Response to Reviewers:

Reviewer 2:

Line 207, modify the spelling of sevier to severe

*We thank the reviewer for their positive constructive feedback. We have*

*corrected the spelling of “sevier” to “severe” in the Methods section under student*

*exclusion criteria.*

Reviewer 3:
1. The study design should be interpreted more cautiously throughout the manuscript.
Although the authors have acknowledged that this is a quasi-experimental pre-post study without a control group, several statements in the Abstract, Discussion, and Conclusion still imply causal effectiveness.
Because no concurrent control group exists, improvements observed after the intervention may reflect
- testing effects,
- Hawthorne effects,
- maturation,
- temporal influences,
- regression to the mean.
I recommend consistently replacing causal wording such as
“the intervention improved…”
with more cautious wording such as
“the intervention was associated with improvements…”
or
“participants demonstrated improvements following the intervention.”
The Abstract is improved but the Discussion still occasionally overstates the findings.

*We agree with the reviewer and have conducted a careful revision*

*across the Abstract, Discussion, and Conclusion sections. We replaced causal*

*phrasing (e.g., “improved”, “led to” ,“caused”) with associative and descriptive*

*terminology (e.g., “was associated with improvements in…”, “demonstrated*

*increase in…”.*

2. Interpretation of the regression model requires revision.
The newly added regression analysis is appreciated.
However, I am not convinced that the interpretation is entirely appropriate.
The model demonstrates that
- baseline social distance strongly predicts post-intervention social distance,
whereas baseline knowledge and attitudes are not independently associated after adjustment.
Yet the Discussion still concludes that
“The more knowledgeable students were about mental illness, the greater the observed reduction in social distance…”
This statement is not supported by the adjusted regression model, since the coefficient for baseline knowledge is non-significant.
The Discussion should instead emphasize that
- baseline stigma appears to be the strongest determinant of post-intervention stigma,
- school context also contributes,
- baseline knowledge alone was not an independent predictor.
This distinction is important.

*We have rewritten the relevant paragraphs in the Results and*

*Discussion sections. Specifically, we removed the statement claiming that higher*

*baseline knowledge predicts lower social distance. Instead, we explicitly state that:*

1. *Baseline social distance is the strongest independent predictor of post-intervention social*

*distance.*

2. *Baseline knowledge and positive attitudes were not independently associated with post-*

*intervention social distance once baseline levels were accounted for.*

3. *School affiliation contributed meaningfully as a covariate.*

3. School-level clustering has not been adequately considered.
Students were nested within only four schools.
Although school affiliation was included as a fixed covariate, the observations are still clustered.
The manuscript should discuss that
- responses within schools may not be statistically independent,
- multilevel modelling was not feasible because only four clusters were available,
- standard errors may therefore be underestimated.
This limitation deserves explicit acknowledgment.

*We appreciate this statistical nuance. We have added a clear discussion of school-*

*level clustering to the Limitations section. We explicitly state that, due to the limited number*

*of school clusters from which data was gathered, multilevel modeling was not*

*methodologically feasible. Additionally, we acknowledged that student responses within the*

*same school may not be fully independent, meaning standard errors could be underestimated.*

4. Sample size justification remains relatively weak.
The revised explanation based on operational feasibility is acceptable.
Nevertheless, the manuscript should explicitly state that
- no formal power calculation was performed,
- the study should be considered exploratory/pilot in nature.
This will improve methodological transparency.

*In the revised manuscript, we updated both the Methods (participants/ sample*

*size) and the Discussion (Limitations section) to clearly state that no formal prior power*

*calculation was performed. We clarified that the study should be interpreted as an*

*exploratory/ pilot implementation trial design to inform future large-scale randomized*

*controlled trials.*

5. External validity should be discussed more explicitly.
The intervention was conducted among
- Grade 9 students,
- urban schools,
- Addis Ababa,
- four selected schools willing to participate.
Therefore, generalizability to
- rural Ethiopia,
- younger adolescents,
- older students,
- out-of-school youth,
is uncertain.
The Discussion should elaborate on this issue.

*We thank the reviewer for this observation. We have expanded our Discussion*

*section to clearly address the boundaries of the study’s external validity. Specifically, we*

*emphasize that since the trial was limited to nineth-grade students in selected urban schools*

*in Addis Ababa, Caution is necessary when generalizing these preliminary findings to rural*

*regions of Ethiopia, younger or older student groups, or out-of-school youth.*

6. 1. Psychometric properties
Cronbach’s alpha values of
- 0.63
- 0.63
- 0.67
are relatively modest.
The Discussion should briefly acknowledge that some translated scales demonstrated only moderate internal consistency and that formal psychometric validation in Ethiopia remains necessary.

*In the Discussion and Limitations sections, we now acknowledge the modest*

*Cronbach’s alpha values (α=0.63-0.67) of these newly translated Amharic instruments. We*

*explain that these values highlight the need for dedicated, formal psychometric validation*

*studies of mental health stigma scales among Ethiopian adolescents.*

7. Clarify missing data
The authors report 14 participants lost to follow-up.
Please clarify whether
- complete-case analysis was performed,
- any comparison between completers and non-completers was undertaken.
*Thank you for giving us the chance to clarify this point. The 14 excluded*

*participants were a mix of students absent on the day of the post-assessment day and students*

*with incomplete or missing data in their digital survey entries. We conducted complete-case*

*analysis, examining the 186 participants who completed both pre- and post-intervention*

*evaluations. No formal statistical comparison analysis was performed between completers*

*and non-completers.*

8. Statistical reporting
The statistical reporting is much improved.
However, I recommend reporting
- exact p-values whenever possible instead of simply “<0.001”,
- particularly within tables.

*Thank you for this suggestion. We have revised our text and tables (Tables 2, 3*

*and 4) to report exact p-values where available. Where p-values were extremely small, we*

*updated them according to the standard journal guideline to p<0.001*

9. Effect size interpretation
Please avoid qualitative interpretations such as
“moderate”
or
“substantial”
without citing the thresholds used.
Simply reporting Cohen’s d and r is sufficient.

*We have removed subjective descriptors such as “moderate” or” substantial” from*

*the Results and Discussion text when presenting effect sizes. Now, the text presents the*

*calculated Cohen’s d and Wilcoxon r values directly alongside their confidence intervals*

*without unreferenced qualitative labels.*

10. Language polishing
The manuscript has improved considerably but would still benefit from careful English editing.
Examples include:
- “week long” → “week-long”
- “train-the trainer” → “train-the-trainer”
- several spacing inconsistencies around citations
- occasional grammatical errors
- inconsistent use of “mental health problems” versus “mental disorders”
A final professional language edit is recommended.

*We have completed a line-by-line language and copy-editing pass across the*

*manuscript. We corrected hyphenation issues, fixed citation spacing, and standardized*

*terminology.*
