# Supplementary material for: From stigma to support: Assessing the impact of school mental health literacy program in Addis Ababa, Ethiopia
Source: PLOS Glob Public Health. 2026 Sep 1;6(9):e0007153. doi: 10.1371/journal.pgph.0007153 (PMC13533382; doi:10.1371/journal.pgph.0007153)
Supplement: Peer review history

# Peer review: Decision Letter 0

**DOI:** 10.1371/journal.pgph.0007153.r001

**Authors:** Katherine Kokkinias

12 Feb 2026

PGPH-D-25-03037

From Stigma to Support: Assessing the Impact of School Mental Health Literacy Programs in Addis Ababa, Ethiopia

PLOS Global Public Health

Dear Dr. Veliaminova,

Thank you for submitting your manuscript to PLOS Global Public Health. After careful consideration, we feel that it has merit but does not fully meet PLOS Global Public Health’s publication criteria as it currently stands. Therefore, we invite you to submit a revised version of the manuscript that addresses the points raised during the review process.

==============================

Please note that we have only been able to secure a single reviewer to assess your manuscript. We are issuing a decision on your manuscript at this point to prevent further delays in the evaluation of your manuscript. Please be aware that the editor who handles your revised manuscript might find it necessary to invite additional reviewers to assess this work once the revised manuscript is submitted. However, we will aim to proceed on the basis of this single review if possible.

The reviewer raised a number of concerns that need attention. They request additional information on methodological aspects of the study (such as the more background information about the SN and HEWs that were trained as well as more detailed information about the presentation). They have provided a suggestion for a figure and recommend more discussion about the challenges of this type of intervention.

Could you please revise the manuscript to carefully address the concerns raised?

==============================

Please submit your revised manuscript by Mar 29 2026 11:59PM. If you will need more time than this to complete your revisions, please reply to this message or contact the journal office at globalpubhealth@plos.org. When you're ready to submit your revision, log on to https://www.editorialmanager.com/pgph/ and select the 'Submissions Needing Revision' folder to locate your manuscript file.

Please include the following items when submitting your revised manuscript:

- A letter that responds to each point raised by the editor and reviewer(s). You should upload this letter as a separate file labeled 'Response to Reviewers'.
- A marked-up copy of your manuscript that highlights changes made to the original version. You should upload this as a separate file labeled 'Revised Manuscript with Track Changes'.
- An unmarked version of your revised paper without tracked changes. You should upload this as a separate file labeled 'Manuscript'.

Guidelines for resubmitting your figure files are available below the reviewer comments at the end of this letter.

We look forward to receiving your revised manuscript.

Kind regards,

Katherine Demi Kokkinias, Ph.D.

Staff Editor

PLOS Global Public Health

Journal Requirements:

1. Please include a complete copy of PLOS’ questionnaire on inclusivity in global research in your revised manuscript. Our policy for research in this area aims to improve transparency in the reporting of research performed outside of researchers’ own country or community. The policy applies to researchers who have travelled to a different country to conduct research, research with Indigenous populations or their lands, and research on cultural artefacts. The questionnaire can also be requested at the journal’s discretion for any other submissions, even if these conditions are not met. Please find more information on the policy and a link to download a blank copy of the questionnaire here: https://journals.plos.org/globalpublichealth/s/best-practices-in-research-reporting. Please upload a completed version of your questionnaire as Supporting Information when you resubmit your manuscript.

2. Please amend your detailed Financial Disclosure statement. This is published with the article. It must therefore be completed in full sentences and contain the exact wording you wish to be published.

i. Please clarify all sources of financial support for your study. List the grants, grant numbers, and organizations that funded your study, including funding received from your institution. Please note that suppliers of material support, including research materials, should be recognized in the Acknowledgements section rather than in the Financial Disclosure.

ii. State the initials, alongside each funding source, of each author to receive each grant. For example: "This work was supported by the National Institutes of Health (####### to AM; ###### to CJ) and the National Science Foundation (###### to AM)."

iii. State what role the funders took in the study. If the funders had no role in your study, please state: “The funders had no role in study design, data collection and analysis, decision to publish, or preparation of the manuscript.”

iv. If any authors received a salary from any of your funders, please state which authors and which funders.

3. Please ensure that your Ethics Statement is available in its entirety at the beginning of your Methods section, under a subheading 'Ethics Statement'.

4. Please upload separate figure files in .tif or .eps format. Also, remove the figures from your manuscript file but keep the legends.

For more information about figure files please see our guidelines:

https://journals.plos.org/globalpublichealth/s/figures

https://journals.plos.org/globalpublichealth/s/figures#loc-file-requirements

5. We have noticed that you have cited Tables 3 and 5 in the manuscript file but there are no corresponding tables in the manuscript. Please amend your manuscript to include this table, noting that tables should not be uploaded as individual files.

6. We have noticed that you have cited ‘supplement 1 and 2’ in your manuscript. However, there are no corresponding files uploaded to the submission. Please upload them as separate files with the item type 'Supporting Information'.

7. In this instance it seems there may be acceptable restrictions in place that prevent the public sharing of your minimal data. However, in line with our goal of ensuring long-term data availability to all interested researchers, PLOS’ Data Policy states that authors cannot be the sole named individuals responsible for ensuring data access (http://journals.plos.org/plosone/s/data-availability#loc-acceptable-data-sharing-methods).

Data requests to a non-author institutional point of contact, such as a data access or ethics committee, helps guarantee long term stability and availability of data. Providing interested researchers with a durable point of contact ensures data will be accessible even if an author changes email addresses, institutions, or becomes unavailable to answer requests.

Before we proceed with your manuscript, please also provide non-author contact information (phone/email/hyperlink) for a data access committee, ethics committee, or other institutional body to which data requests may be sent. If no institutional body is available to respond to requests for your minimal data, please consider if there any institutional representatives who did not collaborate in the study, and are not listed as authors on the manuscript, who would be able to hold the data and respond to external requests for data access? If so, please provide their contact information (i.e., email address). Please also provide details on how you will ensure persistent or long-term data storage and availability.

If the reviewer comments include a recommendation to cite specific previously published works, please review and evaluate these publications to determine whether they are relevant and should be cited. There is no requirement to cite these works unless the editor has indicated otherwise.

Additional Editor Comments (if provided):

[Note: HTML markup is below. Please do not edit.]

Reviewers' comments:

Reviewer's Responses to Questions

**Comments to the Author**

1. Does this manuscript meet PLOS Global Public Health’s publication criteria?

Reviewer #1: Yes

**********

2. Has the statistical analysis been performed appropriately and rigorously?

Reviewer #1: Yes

**********

3. Have the authors made all data underlying the findings in their manuscript fully available (please refer to the Data Availability Statement at the start of the manuscript PDF file)?

The PLOS Data policy

Reviewer #1: Yes

**********

4. Is the manuscript presented in an intelligible fashion and written in standard English?

Reviewer #1: Yes

**********

Reviewer #1: Dear Editor,

I find the purpose of the paper relevant and could add value to the literature on the effectiveness of anti-stigma school-based interventions for mental health. The cultural adaptation of Link and his colleagues’ model of “Eliminating the Stigma of Differences” in Ethiopian context and including three components of the intervention including the contact with the person with mental illness adds strength to the study. The background and the key variables are introduced adequately. The purpose of the study is clearly stated and appropriate methodology and tools are followed.

To further strengthen the paper, I have provided my comments below:

• If I understand correctly, the study design used is “Pre-Post quasi experimental without control design”. But in the methods section it just says pre-post quasi experimental design. Hence, it is better to provide the complete design name.

• Providing a flow-chart of methods will be helpful

• Making the flow of intervention more precise is very criticial. It can also be provided as a supplementary material.

• Need to mention how many SN & HEWs were trained and a bit more background of those personnel.

• Contents of the interventions provided through powerpoint presentations/talks are inadequate, it is important for the readers to know what topics are covered in those talks.

• In the discussion, it will be more appropriate to briefly reflect on the challenges of such interventions based in schools and focusing on mental health stigma reduction.

**********

what does this mean?). If published, this will include your full peer review and any attached files.

**Do you want your identity to be public for this peer review?**  If you choose “no”, your identity will remain anonymous but your review may still be made public.

For information about this choice, including consent withdrawal, please see our Privacy Policy

Reviewer #1: No

**********

[NOTE: If reviewer comments were submitted as an attachment file, they will be attached to this email and accessible via the submission site. Please log into your account, locate the manuscript record, and check for the action link "View Attachments". If this link does not appear, there are no attachment files.]

# Author comment: Author response to Decision Letter 1

**DOI:** 10.1371/journal.pgph.0007153.r002

15 Apr 2026

Attachment Submitted filename: Response to Reviewers.docx

# Peer review: Decision Letter 1

**DOI:** 10.1371/journal.pgph.0007153.r003

**Authors:** Theingi Maung Maung

18 May 2026

PGPH-D-25-03037R1

From Stigma to Support: Assessing the Impact of School Mental Health Literacy Programs in Addis Ababa, Ethiopia

PLOS Global Public Health

Dear Dr. Veliaminova,

Thank you for submitting your manuscript to PLOS Global Public Health. After careful consideration, we feel that it has merit but does not fully meet PLOS Global Public Health’s publication criteria as it currently stands. Therefore, we invite you to submit a revised version of the manuscript that addresses the points raised during the review process.

The paper is generally well organized, and the findings are encouraging.

At the same time, there are several methodological and reporting issues that should be clarified before the manuscript is suitable for publication.

Please submit your revised manuscript by Jul 17 2026 11:59PM. If you will need more time than this to complete your revisions, please reply to this message or contact the journal office at globalpubhealth@plos.org. When you're ready to submit your revision, log on to https://www.editorialmanager.com/pgph/ and select the 'Submissions Needing Revision' folder to locate your manuscript file.

Please include the following items when submitting your revised manuscript:

- A letter that responds to each point raised by the editor and reviewer(s). You should upload this letter as a separate file labeled 'Response to Reviewers'.
- A marked-up copy of your manuscript that highlights changes made to the original version. You should upload this as a separate file labeled 'Revised Manuscript with Track Changes'.
- An unmarked version of your revised paper without tracked changes. You should upload this as a separate file labeled 'Manuscript'.

Guidelines for resubmitting your figure files are available below the reviewer comments at the end of this letter.

As the corresponding author, your ORCID iD is verified in the submission system and will appear in the published article. PLOS supports the use of ORCID, and we encourage all coauthors to register for an ORCID iD and use it as well. Please encourage your coauthors to verify their ORCID iD within the submission system before final acceptance, as unverified ORCID iDs will not appear in the published article. *Only*  the individual author can complete the verification step; PLOS staff *cannot*  verify ORCID iDs on behalf of authors.

We look forward to receiving your revised manuscript.

Kind regards,

Theingi Maung Maung, Ph.D

Academic Editor

PLOS Global Public Health

Journal Requirements:

If the reviewer comments include a recommendation to cite specific previously published works, please review and evaluate these publications to determine whether they are relevant and should be cited. There is no requirement to cite these works unless the editor has indicated otherwise.

Additional Editor Comments (if provided):

[Note: HTML markup is below. Please do not edit.]

Reviewers' comments:

Reviewer's Responses to Questions

**Comments to the Author**

Reviewer #2: (No Response)

Reviewer #3: All comments have been addressed

Reviewer #4: All comments have been addressed

**********

publication criteria?

Reviewer #2: Partly

Reviewer #3: Yes

Reviewer #4: Yes

**********

3. Has the statistical analysis been performed appropriately and rigorously?

Reviewer #2: Yes

Reviewer #3: I don't know

Reviewer #4: Yes

**********

4. Have the authors made all data underlying the findings in their manuscript fully available (please refer to the Data Availability Statement at the start of the manuscript PDF file)?

The PLOS Data policy

Reviewer #2: No

Reviewer #3: Yes

Reviewer #4: No

**********

5. Is the manuscript presented in an intelligible fashion and written in standard English?

Reviewer #2: Yes

Reviewer #3: Yes

Reviewer #4: Yes

**********

Reviewer #2: Suggestions for modification and questions to clarify some statements are provided as follows.

1. The abbreviation should be mentioned for the first time. For example: SNs and HEWs in line 167 instead of line 177, and SWM in line 180.

2. Authors should explain how the planned sample is selected (line 250).

3. No text describes how students' mental health status was assessed. The detailed inclusion and exclusion criteria should be included.

4. In line 219, it is mentioned that a total of 7 SNs and HEWs, but there was a total of 10 staff according to their education level. Therefore, it is necessary to clarify.

5. Subtitles should be written perfectly instead of one word like “Measures” in line 230.

6. Because the Cronbach alpha values are not so good (0.63), is there any removal of original items for the local study before data collection? ( line 239 and line 246)

7. Interactive activities “writing a letter to a person with lived experience” are not clear (line 278), and so easier-to-understand examples should be provided. In this example, what would the author like to say to a person with lived experience? During this activity, how does the investigator deal with the ethical issues, especially regarding confidentiality and the rights of the participants? These issues should be described.

8. Also, what does young adults mean in line 279? How did the author find out about such people who are living with a mental disorder? What is the sampling procedure?

9. Explanation about posters is included twice, with a slogan “I am not my mental illness,” in lines 300 and 313. So these sentences should be modified.

10. How was the sample size determined for this study?

11. Is there any loss to follow up after the intervention?

12. In line 318, the title should be “Cultural adaptation steps for intervention package development” rather than Cultural Adaptation.

13. In line 342, the authors mention Spearman's Rank Correlation, but in the results section, no description is found for this analysis.

14. A working definition of “school affiliation” should be included. (line 408)

15. Is there any multicollinearity check before linear regression, because independent variables may be linked to each other, like the knowledge and attitude scale with knowledge and attitude change, and the school affiliation of different schools? (for Table 3)

16. In line 566, there is a discussion point regarding education level referencing Girma et al., 2013. In the current study, there is no consideration of the education level as a predictor variable in the analysis phase, and so this discussion is not aligned with the findings.

17. A label should be included for the study flow diagram.

18. Line spacing and alignments should be modified to meet the Journal format.

Reviewer #3: Thank you for the opportunity to review this manuscript. The topic is highly relevant to global public health, particularly in low- and middle-income countries where adolescent mental health services and stigma reduction programs remain limited. The manuscript addresses an important gap by evaluating a culturally adapted school-based mental health literacy intervention in Ethiopia. I particularly appreciate the authors’ efforts to culturally adapt the intervention and involve local stakeholders and adolescents in the process. The paper is generally well organized, and the findings are encouraging.

At the same time, there are several methodological and reporting issues that should be clarified before the manuscript is suitable for publication.

1. Study design and interpretation of findings

The main limitation of the study is the absence of a control group. Since this is a pre-post quasi-experimental study without comparison participants, it is difficult to determine how much of the observed improvement can truly be attributed to the intervention itself. Some of the wording in the Results and Discussion sections implies causality or effectiveness more strongly than the design can support.

I would encourage the authors to moderate some of the conclusions and frame the findings more cautiously, for example by stating that the intervention was “associated with improvements” rather than definitively “demonstrated effectiveness.”

The limitation is acknowledged later in the manuscript, but it should be emphasized more clearly when interpreting the results.

2. More detail is needed regarding participant recruitment and follow-up

The manuscript would benefit from clearer reporting of participant flow.

The authors mention that 200 students were targeted, while the final analyses included 186 participants. It would help readers to know:

- how many students declined participation,

- whether there was any loss to follow-up,

- whether missing data occurred,

- and whether all students completed both pre- and post-intervention assessments.

A simple participant flow diagram would improve transparency considerably.

In addition, more explanation regarding how the four schools were selected would strengthen the manuscript.

3. Psychometric properties of the scales

Several scales showed relatively low Cronbach alpha values, particularly around 0.63–0.67. While this may be understandable in cross-cultural adolescent research, the implications should be discussed more carefully.

Since cultural adaptation is a major strength of this study, it would be helpful to provide more information on:

- whether pilot testing was conducted,

- whether adolescents had difficulty understanding any items,

- and whether the translated instruments were formally validated in the Ethiopian context.

4. Statistical analysis needs clarification

The statistical approach is generally appropriate, but several parts need clearer explanation.

First, the rationale for using Wilcoxon signed-rank tests for the binary-response scales should be explained more explicitly.

Second, the regression model predicting post-intervention social distance would benefit from further clarification. Including both baseline scores and change scores in the same model may introduce statistical dependency. The authors should explain why this approach was chosen and whether multicollinearity was assessed.

5. 5. Intervention implementation and fidelity

The intervention appears thoughtfully designed, especially the combination of classroom teaching, contact-based sessions, and printed materials. However, more detail is needed regarding implementation consistency across schools.

For example:

- Were all sessions delivered in the same way?

- Was fidelity formally monitored?

- Were trainers observed or supervised using a checklist?

- Did the researchers assess variability between schools or trainers?

Given that multiple nurses and health extension workers delivered the program, this information is important.

6. Social desirability bias should be discussed more fully

Most outcomes relied on self-reported attitudes and intentions, which may be influenced by social desirability bias, especially shortly after a mental health awareness intervention.

This is particularly relevant because:

- students were aware of the study objectives,

- the intervention occurred within schools,

- and post-testing was conducted only three weeks later.

I recommend expanding this point in the limitations section.

7. Language and grammar

The manuscript is generally understandable, but there are several grammatical and typographical issues throughout the paper that should be corrected during revision. Examples include:

- “one in meets the criteria”

- “0.04 psychiatrists per 100,000 peoples”

- “theses scores” instead of “these scores”

A careful language edit would improve readability.

8. Strength of the manuscript

One of the strongest aspects of this work is the attention given to cultural adaptation and youth participation. This adds substantial value to the study and makes the intervention more relevant for LMIC settings. I encourage the authors to emphasize this practical contribution further in the Discussion.

Reviewer #4: Overall Assessment

This manuscript addresses an important issue in global mental health, namely the reduction of mental health stigma among adolescents in low- and middle-income countries through school-based mental health literacy interventions. The topic is timely and relevant, particularly given the limited mental health resources and low mental health literacy reported in many LMIC settings. The cultural adaptation of the intervention for the Ethiopian context and the inclusion of a contact-based component are notable strengths.

The reported improvements in mental health knowledge, attitudes toward individuals with mental illness, social distance, and help-seeking intentions are encouraging and broadly consistent with previous research on stigma-reduction interventions among adolescents.

However, several substantial methodological and reporting limitations limit the interpretability of the findings. In its current form, the manuscript does not yet provide sufficiently rigorous evidence to support the conclusions presented. Major revisions would be required to address the issues outlined below.

Major Comments

1. Study design and causal interpretation

The study uses a pre–post design without a comparison or control group. While such designs may provide preliminary evidence regarding potential intervention effects, they do not allow causal attribution of observed changes to the intervention itself.

Several alternative explanations remain possible, including:

• temporal or maturation effects

• social desirability bias

• exposure to external influences during the study period

Although the manuscript reports statistically significant improvements across several outcomes, these findings should be interpreted as associations observed following the intervention, rather than evidence of intervention effectiveness.

The Discussion and Conclusion sections should be revised to avoid causal language and more explicitly acknowledge the limitations of the study design.

2. Short follow-up period

The post-intervention assessment was conducted three weeks after the intervention, which represents a relatively short follow-up period for evaluating changes in attitudes and stigma-related behaviors.

It is therefore unclear whether the observed improvements in knowledge, attitudes, social distance, and help-seeking intentions are sustained over time. The authors should discuss this limitation more explicitly and suggest that future studies incorporate longer follow-up periods to evaluate the durability of intervention effects.

3. Statistical reporting and analytical transparency

The statistical reporting would benefit from additional detail to improve methodological transparency. In particular, the manuscript should consider including:

• confidence intervals for key estimates

• information on the reliability or internal consistency of the measurement scales used (e.g., Cronbach’s alpha)

• clarification of whether clustering at the school level was considered in the analysis

Given that participants were recruited from schools, potential intra-cluster correlation may influence variance estimates and should be addressed or acknowledged.

4. Data availability and journal policy compliance

The Data Availability Statement indicates that the data are available only upon reasonable request due to ethical restrictions. However, journals such as PLOS typically require that all underlying data supporting the results be publicly available, unless there are clear ethical or legal constraints.

The authors should clarify whether a de-identified dataset could be deposited in a public repository or provided as supplementary material in accordance with journal data-sharing requirements.

5. Intervention description and reproducibility

The manuscript would benefit from a more detailed description of the intervention and its cultural adaptation process.

Specifically, the authors should provide additional information on:

• how the intervention was culturally adapted for the Ethiopian context

• the training and qualifications of facilitators

• the duration and structure of each intervention component

Providing these details would improve the reproducibility and external applicability of the intervention for other LMIC settings.

Concerns Regarding Interpretation of Results

Overinterpretation in the Conclusion

The current conclusion appears to overstate the strength of the evidence relative to the study design.

Example (Lines 656–658)

“This study demonstrated successful reduction in stigma toward individuals with mental health problems and improvements in mental health knowledge and help-seeking behavior…”

This statement implies demonstrated effectiveness, which is not supported by the current study design. A more appropriate interpretation would be that improvements were observed following the intervention, rather than that the intervention was proven to be effective.

Example (Lines 659–661)

“Such interventions can enhance mental health knowledge, reduce stigma and potentially improve …”

This sentence appears to generalize the findings beyond the study population. Conclusions should remain closely aligned with the evidence generated from the current study.

Introduction: Clarification of the Knowledge Gap

While the Introduction provides relevant background on mental health stigma and adolescent mental health literacy in LMICs, the specific research gap remains insufficiently articulated.

The authors should more clearly:

• synthesize existing evidence on school-based anti-stigma interventions in LMICs, and

• explicitly state what gap in the literature this study aims to address.

Clarifying this point would strengthen the conceptual positioning of the study.

Recommendation

While the manuscript addresses an important topic in global mental health and presents promising preliminary findings, the current methodological limitations and issues in interpretation substantially weaken the strength of the evidence presented.

At present, the manuscript does not yet meet the methodological and reporting standards required for publication, and substantial revision would be necessary to address the concerns outlined above.

**********

what does this mean?). If published, this will include your full peer review and any attached files.

**Do you want your identity to be public for this peer review?**  If you choose “no”, your identity will remain anonymous but your review may still be made public.

For information about this choice, including consent withdrawal, please see our Privacy Policy

Reviewer #2: **Yes:**  May Soe Aung, University of Medicine (1) , Myanmar

Reviewer #3: No

Reviewer #4: No

**********

[NOTE: If reviewer comments were submitted as an attachment file, they will be attached to this email and accessible via the submission site. Please log into your account, locate the manuscript record, and check for the action link "View Attachments". If this link does not appear, there are no attachment files.]

# Author comment: Author response to Decision Letter 2

**DOI:** 10.1371/journal.pgph.0007153.r004

9 Jul 2026

Attachment Submitted filename: Response_to_Reviewers_auresp_2.docx

# Peer review: Decision Letter 2

**DOI:** 10.1371/journal.pgph.0007153.r005

**Authors:** Theingi Maung Maung

20 Jul 2026

PGPH-D-25-03037R2

From Stigma to Support: Assessing the Impact of School Mental Health Literacy Programs in Addis Ababa, Ethiopia

PLOS Global Public Health

Dear Dr. Veliaminova,

Thank you for submitting your manuscript to PLOS Global Public Health. After careful consideration, we feel that it has merit but does not fully meet PLOS Global Public Health’s publication criteria as it currently stands. Therefore, we invite you to submit a revised version of the manuscript that addresses the points raised during the review process.

Overall, the manuscript has improved substantially in terms of methodological transparency, statistical reporting, and presentation of the intervention. We still have several comments on study design, interpretation, sample size justification that should be addressed before publication.

Please submit your revised manuscript by Aug 19 2026 11:59PM. If you will need more time than this to complete your revisions, please reply to this message or contact the journal office at globalpubhealth@plos.org. When you're ready to submit your revision, log on to https://www.editorialmanager.com/pgph/ and select the 'Submissions Needing Revision' folder to locate your manuscript file.

Please include the following items when submitting your revised manuscript:

- A letter that responds to each point raised by the editor and reviewer(s). You should upload this letter as a separate file labeled 'Response to Reviewers'.
- A marked-up copy of your manuscript that highlights changes made to the original version. You should upload this as a separate file labeled 'Revised Manuscript with Track Changes'.
- An unmarked version of your revised paper without tracked changes. You should upload this as a separate file labeled 'Manuscript'.

Guidelines for resubmitting your figure files are available below the reviewer comments at the end of this letter.

As the corresponding author, your ORCID iD is verified in the submission system and will appear in the published article. PLOS supports the use of ORCID, and we encourage all coauthors to register for an ORCID iD and use it as well. Please encourage your coauthors to verify their ORCID iD within the submission system before final acceptance, as unverified ORCID iDs will not appear in the published article. *Only*  the individual author can complete the verification step; PLOS staff *cannot*  verify ORCID iDs on behalf of authors.

We look forward to receiving your revised manuscript.

Kind regards,

Theingi Maung Maung, Ph.D

Academic Editor

PLOS Global Public Health

Journal Requirements:

If the reviewer comments include a recommendation to cite specific previously published works, please review and evaluate these publications to determine whether they are relevant and should be cited. There is no requirement to cite these works unless the editor has indicated otherwise.

Please review your reference list to ensure that it is complete and correct. If you have cited papers that have been retracted, please include the rationale for doing so in the manuscript text, or remove these references and replace them with relevant current references. Any changes to the reference list should be mentioned in the rebuttal letter that accompanies your revised manuscript. If you need to cite a retracted article, indicate the article’s retracted status in the References list and also include a citation and full reference for the retraction notice.

Additional Editor Comments (if provided):

[Note: HTML markup is below. Please do not edit.]

Reviewers' comments:

Reviewer's Responses to Questions

**Comments to the Author**

Reviewer #2: All comments have been addressed

Reviewer #3: All comments have been addressed

**********

publication criteria?

Reviewer #2: Yes

Reviewer #3: Partly

**********

3. Has the statistical analysis been performed appropriately and rigorously?

Reviewer #2: Yes

Reviewer #3: No

**********

4. Have the authors made all data underlying the findings in their manuscript fully available (please refer to the Data Availability Statement at the start of the manuscript PDF file)?

The PLOS Data policy

Reviewer #2: Yes

Reviewer #3: Yes

**********

5. Is the manuscript presented in an intelligible fashion and written in standard English?

Reviewer #2: Yes

Reviewer #3: Yes

**********

Reviewer #2: Authors adressed all comments from reviewer. Thank you for your detailed reply.

Only one suggestion is provided for spellin mistake "Line 207 To modify the spelling of sevier to severe".

Reviewer #3: Overall, the manuscript has improved substantially in terms of methodological transparency, statistical reporting, and presentation of the intervention. The additional details regarding cultural adaptation, implementation procedures, effect sizes, confidence intervals, and regression analyses have strengthened the scientific rigor of the study.

The topic remains highly relevant for global public health, particularly given the scarcity of evidence on school-based mental health stigma interventions in low- and middle-income countries. The manuscript provides valuable preliminary evidence that a culturally adapted intervention may improve mental health literacy and reduce stigma among adolescents in Ethiopia.

Although the revision is much improved, I still have several comments that should be addressed before publication.

1. The study design should be interpreted more cautiously throughout the manuscript.

Although the authors have acknowledged that this is a quasi-experimental pre-post study without a control group, several statements in the Abstract, Discussion, and Conclusion still imply causal effectiveness.

Because no concurrent control group exists, improvements observed after the intervention may reflect

- testing effects,

- Hawthorne effects,

- maturation,

- temporal influences,

- regression to the mean.

I recommend consistently replacing causal wording such as

“the intervention improved…”

with more cautious wording such as

“the intervention was associated with improvements…”

or

“participants demonstrated improvements following the intervention.”

The Abstract is improved but the Discussion still occasionally overstates the findings.

2. Interpretation of the regression model requires revision.

The newly added regression analysis is appreciated.

However, I am not convinced that the interpretation is entirely appropriate.

The model demonstrates that

- baseline social distance strongly predicts post-intervention social distance,

whereas baseline knowledge and attitudes are not independently associated after adjustment.

Yet the Discussion still concludes that

“The more knowledgeable students were about mental illness, the greater the observed reduction in social distance…”

This statement is not supported by the adjusted regression model, since the coefficient for baseline knowledge is non-significant.

The Discussion should instead emphasize that

- baseline stigma appears to be the strongest determinant of post-intervention stigma,

- school context also contributes,

- baseline knowledge alone was not an independent predictor.

This distinction is important.

3. School-level clustering has not been adequately considered.

Students were nested within only four schools.

Although school affiliation was included as a fixed covariate, the observations are still clustered.

The manuscript should discuss that

- responses within schools may not be statistically independent,

- multilevel modelling was not feasible because only four clusters were available,

- standard errors may therefore be underestimated.

This limitation deserves explicit acknowledgment.

4. Sample size justification remains relatively weak.

The revised explanation based on operational feasibility is acceptable.

Nevertheless, the manuscript should explicitly state that

- no formal power calculation was performed,

- the study should be considered exploratory/pilot in nature.

This will improve methodological transparency.

5. External validity should be discussed more explicitly.

The intervention was conducted among

- Grade 9 students,

- urban schools,

- Addis Ababa,

- four selected schools willing to participate.

Therefore, generalizability to

- rural Ethiopia,

- younger adolescents,

- older students,

- out-of-school youth,

is uncertain.

The Discussion should elaborate on this issue.

6. 1. Psychometric properties

Cronbach’s alpha values of

- 0.63

- 0.63

- 0.67

are relatively modest.

The Discussion should briefly acknowledge that some translated scales demonstrated only moderate internal consistency and that formal psychometric validation in Ethiopia remains necessary.

7. Clarify missing data

The authors report 14 participants lost to follow-up.

Please clarify whether

- complete-case analysis was performed,

- any comparison between completers and non-completers was undertaken.

8. Statistical reporting

The statistical reporting is much improved.

However, I recommend reporting

- exact p-values whenever possible instead of simply “<0.001”,

- particularly within tables.

9. Effect size interpretation

Please avoid qualitative interpretations such as

“moderate”

or

“substantial”

without citing the thresholds used.

Simply reporting Cohen’s d and r is sufficient.

10. Language polishing

The manuscript has improved considerably but would still benefit from careful English editing.

Examples include:

- “week long” → “week-long”

- “train-the trainer” → “train-the-trainer”

- several spacing inconsistencies around citations

- occasional grammatical errors

- inconsistent use of “mental health problems” versus “mental disorders”

A final professional language edit is recommended.

**********

what does this mean?). If published, this will include your full peer review and any attached files.

**Do you want your identity to be public for this peer review?**  If you choose “no”, your identity will remain anonymous but your review may still be made public.

For information about this choice, including consent withdrawal, please see our Privacy Policy

Reviewer #2: **Yes:**  Dr. May Soe Aung, Associate Professor, Department of Preventive and Social Medicine, University of Medicine (1), Yangon, Myanmar

Reviewer #3: No

**********

[NOTE: If reviewer comments were submitted as an attachment file, they will be attached to this email and accessible via the submission site. Please log into your account, locate the manuscript record, and check for the action link "View Attachments". If this link does not appear, there are no attachment files.]

# Author comment: Author response to Decision Letter 3

**DOI:** 10.1371/journal.pgph.0007153.r006

3 Aug 2026

Attachment Submitted filename: Response_to_Reviewers_auresp_3.docx

# Editor report: Decision Letter 3

**DOI:** 10.1371/journal.pgph.0007153.r007

**Authors:** Theingi Maung Maung

5 Aug 2026

From Stigma to Support: Assessing the Impact of School Mental Health Literacy Programs in Addis Ababa, Ethiopia

PGPH-D-25-03037R3

Dear Mrs Veliaminova,

We are pleased to inform you that your manuscript 'From Stigma to Support: Assessing the Impact of School Mental Health Literacy Programs in Addis Ababa, Ethiopia ' has been provisionally accepted for publication in PLOS Global Public Health.

Before your manuscript can be formally accepted you will need to complete some formatting changes, which you will receive in a follow up email. A member of our team will be in touch with a set of requests.

Please note that your manuscript will not be scheduled for publication until you have made the required changes, so a swift response is appreciated.

IMPORTANT: The editorial review process is now complete. PLOS will only permit corrections to spelling, formatting or significant scientific errors from this point onwards. Requests for major changes, or any which affect the scientific understanding of your work, will cause delays to the publication date of your manuscript.

If your institution or institutions have a press office, please notify them about your upcoming paper to help maximize its impact. If they'll be preparing press materials, please inform our press team as soon as possible -- no later than 48 hours after receiving the formal acceptance. Your manuscript will remain under strict press embargo until 2 pm Eastern Time on the date of publication. For more information, please contact globalpubhealth@plos.org.

Thank you again for supporting Open Access publishing; we are looking forward to publishing your work in PLOS Global Public Health.

Best regards,

Theingi Maung Maung, Ph.D

Academic Editor

PLOS Global Public Health

***********************************************************

Reviewer Comments (if any, and for reference):
